# Selective activation of tetrodotoxin-sensitive sodium channels by AaH-II drives arrhythmogenic late Na^+^ current in the heart

**DOI:** 10.1093/europace/euag249

**Published:** 2026-09-03

**Authors:** Hugo Millet, Maureen Choteau-Bodor, Thomas Stervinou, Floriane Bibault, Sarah Godin, Pauline Belhumeur, Matthias Dereli, Agnès Hivonnait, Morteza Erfanian, Agnès Tessier, Aurore Girardeau, Guillaume Lamirault, Flavien Charpentier, Mikael Croyal, Nathalie Gaborit, Jérôme Montnach, Michel De Waard

**Affiliations:** Nantes Université, CNRS, INSERM, l’institut du thorax, Nantes F-44000, France; Nantes Université, CNRS, INSERM, l’institut du thorax, Nantes F-44000, France; Nantes Université, CNRS, INSERM, l’institut du thorax, Nantes F-44000, France; Nantes Université, CNRS, INSERM, l’institut du thorax, Nantes F-44000, France; Nantes Université, CNRS, INSERM, l’institut du thorax, Nantes F-44000, France; Nantes Université, CNRS, INSERM, l’institut du thorax, Nantes F-44000, France; Nantes Université, CNRS, INSERM, l’institut du thorax, Nantes F-44000, France; Nantes Université, CNRS, INSERM, l’institut du thorax, Nantes F-44000, France; Nantes Université, CNRS, INSERM, l’institut du thorax, Nantes F-44000, France; Nantes Université, CNRS, INSERM, l’institut du thorax, Nantes F-44000, France; Nantes Université, CNRS, INSERM, l’institut du thorax, Nantes F-44000, France; Nantes Université, CNRS, INSERM, l’institut du thorax, Nantes F-44000, France; Nantes Université, CNRS, INSERM, l’institut du thorax, Nantes F-44000, France; Nantes Université, CNRS, INSERM, l’institut du thorax, Nantes F-44000, France; Nantes Université, CHU Nantes, Inserm, CNRS, SFR Santé, Inserm UMS 016, CNRS UMS 3556, Nantes, France; Nantes Université, CNRS, INSERM, l’institut du thorax, Nantes F-44000, France; Nantes Université, CNRS, INSERM, l’institut du thorax, Nantes F-44000, France; Nantes Université, CNRS, INSERM, l’institut du thorax, Nantes F-44000, France; Smartox Biotechnology, 3B rue de l'Isère, 38120 Saint Egrève, France

**Keywords:** Late sodium current (I_NaL_), TTX-sensitive sodium channels, AaH-II peptide, Cardiac arrhythmia, Scorpion venom peptide

## Abstract

Increased late sodium current (I_NaL_) is a central mechanism underlying both inherited and acquired cardiac arrhythmias. Although Na_v_1.5 is the dominant cardiac sodium channel, multiple tetrodotoxin-sensitive (TTX-S) Na_v_ isoforms are also expressed in cardiomyocytes, particularly within transverse tubules, where they influence excitation-contraction coupling. Whether these channels contribute directly to pathological I_NaL_ and arrhythmogenesis remains unresolved, in part because available pharmacological tools lack isoform selectivity. Herein, our objective was to determine the contribution of TTX-S Na_v_ channels to pathological cardiac I_NaL_ and arrhythmia and to establish selective pharmacological tools to dissect their role. Using automated patch-clamp and human Na_v_ isoform profiling, we show that the reference I_NaL_ inducer ATX-II predominantly activates TTX-resistant Na_v_1.5 Na_v_ channels. However, ATX-II lacks Na_v_ isoform selectivity when inappropriately used, questioning the conclusions reached by numerous cardiac I_NaL_ studies. In contrast, AaH-II, a peptide from *Androctonus australis hector* scorpion venom, more selectively and potently enhances I_NaL_ through TTX-S Na_v_ isoforms. In human iPS-derived cardiomyocytes, TTX-S I_NaL_ leads to action potential prolongation. In adult ventricular cardiomyocytes, AaH-II induces abnormal Ca^2+^ handling and spontaneous Ca^2+^ release events. In isolated hearts and *in vivo*, selective TTX-S I_NaL_ activation produces conduction abnormalities, QT prolongation, and ventricular proarrhythmic events, which are prevented by nanomolar tetrodotoxin concentrations that spare Na_v_1.5. These findings demonstrate that TTX-S sodium channels are sufficient to generate arrhythmogenic late Na^+^ current in the heart, independently of Na_v_1.5. AaH-II provides a powerful new tool to selectively probe TTX-S I_NaL_ and reveals these channels as previously underappreciated contributors to cardiac electrical instability. Targeting TTX-S Na_v_ channels may therefore represent a novel and potentially safer strategy for antiarrhythmic therapy.

## Graphical Abstract

**Graphical Abstract:**
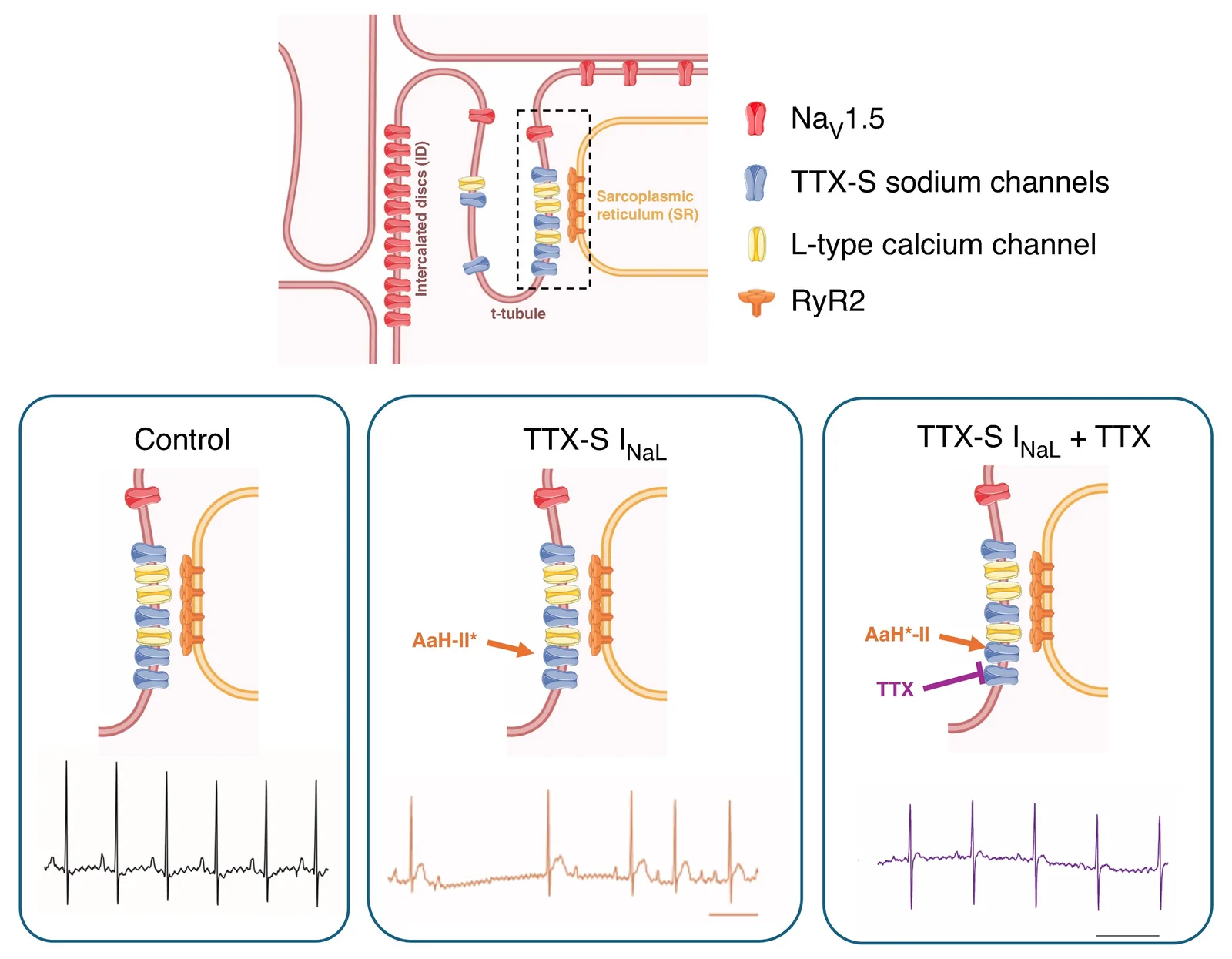

What’s new

- Tetrodotoxin-sensitive (TTX-S) sodium channels are sufficient to generate arrhythmogenic late Na^+^ current in the heart, independently of the canonical cardiac channel Na_v_1.5.
- The widely used reference peptide ATX-II lacks isoform selectivity under the usual conditions of use, activating both TTX-resistant and TTX-S Na_v_ channels, necessitating a reinterpretation of decades of cardiac I_NaL_ studies.
- AaH-II is identified as a high-affinity and functionally selective activator of TTX-S cardiac sodium channels, providing a novel pharmacological tool to dissect isoform-specific late Na^+^ current.
- Selective enhancement of TTX-S I_NaL_ disrupts Ca^2+^ handling and triggers ventricular proarrhythmic events under conditions where Na_v_1.5-mediated late current is negligible.
- TTX-S sodium channels emerge as previously underappreciated and potentially safer antiarrhythmic targets, with implications for drug discovery and cardiac safety pharmacology.

## Introduction

In the heart, the rapid upstroke of the action potential (AP) is mediated by voltage-gated sodium (Na_v_) channels, which in turn activate L-type calcium channels to initiate excitation-contraction coupling through ryanodine receptor opening and sarcoplasmic reticulum (SR) Ca^2+^ release. The precise temporal coordination of these ionic fluxes is essential for synchronous myocardial contraction and normal cardiac rhythm. Repolarization, driven largely by voltage-gated potassium (K_v_) channels, finely terminates the AP, ensuring electrical stability. Disruption of this balance—whether by genetic variation, disease remodelling, or pharmacological intervention—creates the substrate for cardiac arrhythmias. A prominent example of such disruption is congenital long QT syndrome type 3 (LQT3), in which variants in *SCN5A* produce Na_v_1.5 channels with impaired inactivation, giving rise to a persistent or ‘late’ Na^+^ current (late I_Na_, I_NaL_).^1^ This pathological current prolongs the AP plateau, delays repolarization, and promotes proarrhythmic electrical instability. Importantly, increased I_NaL_ is not restricted to rare genetic disorders but also emerges in common acquired conditions such as heart failure and ischaemic heart disease and can be induced by drugs—hence its central role in the Comprehensive in Vitro Proarrhythmia Assay (CiPA) initiative. Despite its clinical relevance, I_NaL_ is intrinsically difficult to study under physiological conditions because it represents less than 0.1% of the total cardiac Na^+^ current. To overcome this limitation, the sea anemone peptide ATX-II, a potent activator of I_NaL_, has been adopted as a reference tool by the U.S. Food and Drug Administration. ATX-II-induced enhancement of I_NaL_ recapitulates key arrhythmogenic mechanisms, including prolonged Ca^2+^ channel opening, intracellular Na^+^ accumulation, SR Ca^2+^ overload, CaMKII activation, ryanodine receptor leak, increased Na^+^/Ca^2+^ exchanger activity, and the emergence of early afterdepolarizations. Consistent with this, ATX-II promotes atrial fibrillation and ventricular arrhythmias in experimental models.^2^ Using this paradigm, compounds such as ranolazine and GS-458967,^3^ as well as several natural products, have been identified as I_NaL_ inhibitors with antiarrhythmic potential.

However, a critical unresolved issue remains: which cardiac Na_v_ channel isoforms are responsible for pathological I_NaL_? Although Na_v_1.5 is the dominant cardiac Na_v_ channel, multiple tetrodotoxin-sensitive (TTX-S) isoforms—including Na_v_1.1, 1.2, 1.3, 1.4, 1.6, and 1.7—are also expressed in cardiomyocytes, alongside the TTX-resistant Na_v_1.8.^4–6^ These channels exhibit striking species- and compartment-specific distributions. In several species, TTX-S channels are enriched within transverse (t)-tubules, whereas Na_v_1.5 is concentrated at intercalated discs,^6–10^ suggesting that TTX-S channels play a disproportionate role in excitation-contraction coupling and local Ca^2+^ signalling despite contributing less to total Na^+^ influx. Indeed, TTX-S channels account for up to 44% of I_NaL_ in canine ventricular myocytes,^11^ and cardiac arrhythmias are frequently observed in neurological and neuromuscular disorders associated with TTX-S channel dysfunction.^12^ Conversely, their apparent dispensability for basal excitation-contraction coupling in some species^13^ raises the intriguing possibility that TTX-S channels could represent safer antiarrhythmic targets than Na_v_1.5. At present, the experimental dissection of TTX-S vs. TTX-resistant Na^+^ channel contributions relies almost exclusively on two imperfect tools: TTX itself, which blocks TTX-S channels below 100 nM while sparing Na_v_1.5, and ATX-II, whose isoform selectivity has never been rigorously defined. This represents a major conceptual and practical gap, as it is essential to know whether the pathological I_NaL_ induced by ATX-II originates solely from Na_v_1.5 or also from TTX-S channels.

Here, we address this gap directly. We establish a standardized, quantitative selectivity profile of ATX-II across human Na_v_ isoforms and identify AaH-II, a peptide from *Androctonus australis hector* scorpion venom, as a high-affinity and selective activator of cardiac TTX-S I_NaL_. We show that preferential activation of TTX-S-mediated I_NaL_ is largely proarrhythmic under conditions in which Na_v_1.5-dependent I_NaL_ is negligible. These findings establish AaH-II as a powerful complementary tool to ATX-II for dissecting the isoform-specific mechanisms of pathological late Na^+^ current and for accelerating the discovery of antiarrhythmic therapies targeting non-Na_v_1.5 Na_v_ channels.

## Methods

### Peptides and compounds

All the peptides used in this study (AaH-II, AaH-II*, and ATX-II) were synthesized by Smartox Biotechnology (https://www.smartox-biotech.com/). AaH-II* is a single amino-acid substituted analogue of AaH-II (R^62^K mutation), previously developed to produce a photoactivable Na_v_ agonist.^14^ As shown on representative hNa_v_1.2 currents, AaH-II and AaH-II* are fully similar in their properties for inducing I_NaL_ (see Supplementary material online, *Figure S1*). Interestingly, however, AaH-II* differs from AaH-II in lacking the inhibitory effect of AaH-II at the highest concentrations, which is presumably due to a second binding site on voltage-sensor domain I that we presume is lost for some Na_v_ isoforms upon this R to K substitution.^15^ TTX was purchased from Tocris Bioscience.

### Ethical statement

All animal care and experimental procedures were performed in the animal facility of *Nantes Université* (UTE IRS-UN), which has been accredited by the French Ministry of Agriculture. Wistar Han 9- to 13-week-old rats were used for *ex vivo* and *in vivo* experiments. All animals were exposed to 12 h light/dark cycles (light, 8:00 AM to 20:00 PM) in a thermostatically controlled room with free access to food and water. The experimental procedures were approved by the regional ethics committee (CEEA-006 Pays de la Loire, France) and authorized by the French Ministry of National Education (APAFIS #34541-2022010310194375), Higher Education and Research, according to Directive 2010/63/EU of the European Union.

### Cell culture

HEK293 cells stably expressing human (h)Na_v_1.1 (NM_001165963.4), hNa_v_1.2 (NM_001040142.2), hNa_v_1.3 (NM_001081676.2), hNa_v_1.5 (NM_000335.5), hNa_v_1.6 (NM_001177984.3), and CHO cells stably expressing Na_v_1.7 (NM_001365536.1) were cultured in Dulbecco’s Modified Eagle’s Medium (DMEM) supplemented with 10% fetal bovine serum, 1 mM pyruvic acid, 4.5 g/L glucose, 4 mM glutamine, 10 U/mL penicillin, and 10 μg/mL streptomycin (Gibco, Grand Island, NY). HEK293 cells stably expressing human Na_v_1.4 (NM_000334.4) were cultured in Minimum Essential Medium (MEM) supplemented with 10% fetal bovine serum, 1 mM pyruvic acid, 4.5 g/L glucose, 4 mM glutamine, 10 U/mL penicillin, and 10 μg/mL streptomycin (Gibco, Grand Island, NY). All cell lines were incubated at 37°C in a 5% CO_2_ atmosphere. G418 (400–800 µg/mL) was used to maintain stable expression of hNa_v_1.1, hNa_v_1.2, hNa_v_1.3, hNa_v_1.4, hNa_v_1.5, and hNa_v_1.6, whereas hygromycin B (200 µg/mL) was used to maintain hNa_v_1.7 expression. Cells prepared for electrophysiological experiments were first detached using trypsin, and isolated cells were diluted (∼300 000 cells/mL) in medium containing (in mM) 140 NaCl, 4 KCl, 2 CaCl_2_, 1 MgCl_2_, 5 glucose, and 10 HEPES (pH 7.4, osmolarity 298 mOsm).

### Human iPS cell culture

The control hiPSC cell line (ITXi002-A; IRX5-Wt^16^; RRID:CVCL_B5QD) was generated from primary dermal fibroblasts and previously characterized.^17–19^ hiPSCs were maintained at 37°C, 5% CO_2_, 21% O_2_ in StemMacs PSC-Brew XF human medium (Miltenyi Biotec, #130-127-865) on culture plates coated with 0.05 mg/mL Matrigel hESC-Qualified Matrix (Corning, #354277) in DMEM F12 (Gibco, #11320033). At 75% confluency, the cells were passaged using the Gentle Cell Dissociation Reagent (StemCell Technologies, #07174).

### Cardioid generation, dissociation, and cell preparation for manual and automated patch-clamp

We generated right ventricular-like cardioids on 96-well low-attachment plates using the Schmidt *et al.* (2023) protocol.^20^ At D15.5, cardioids were pooled, dissociated, and replated as a monolayer in MW12 plates (Falcon, #353047) coated with Matrigel (Corning, #354277) for automated patch-clamp, and as single cells in 35-mm Petri dishes (NUNC #055055) coated with Matrigel for manual patch-clamp. Immediately before patch-clamp recording, cardiomyocytes were single-cell dissociated using Accutase (Sigma, #A6964).

### qRT-PCR experiments for Na_v_ isoform quantification in human iPS-derived cardiomyocytes

At D22.5, 1–2 MW12 wells were rinsed with 1 mL PBS, and then cells were lysed with 350 µL of RA1 buffer from the NucleoSpinTM RNA kit (Macherey Nagel, #740955). RNA extraction was then performed according to the kit’s protocol, with a 60 µL elution in nuclease-free H_2_O. The High-Capacity cDNA Reverse Transcription Kit (Applied Biosystems #4368814) was used according to the manufacturer’s specifications to obtain cDNA from 1 μg of RNA samples. For the qPCR, the TaqMan® Universal PCR Master Mix (Applied Biosystems #4304437) was used with the following primers: *SCN1A* (TaqMan® Hs00374696_m), *SCN2A* (TaqMan® Hs01109871_m), *SCN3A* (TaqMan® Hs00366902_m), *SCN4A* (TaqMan® Hs01109480_m1), *SCN5A* (TaqMan® Hs00165693_m1), *SCN8A* (TaqMan® Hs00274075_m), *SCN9A* (TaqMan® Hs00161567_m1), *SCN10A* (TaqMan® Hs01045137_m1), and *ACTB* (TaqMan® Hs01060665_g1). Raw data were analysed with QuantStudio5. The results were expressed as Ct values, normalized to ACTB expression (dCt) and then graphed as 2^-dCt * 100.

### High-throughput automated patch-clamp

Automated patch-clamp recordings were performed with the SyncroPatch 384PE from Nanion (München, Germany) to investigate the efficacy and/or selectivity of AaH-II, AaH-II*, ATX-II, and TTX on sodium currents. Chips with single-hole medium resistance of 4.51 ± 0.01 MΩ (*n* = 384) were used for recordings. Pulse generation and data collection were performed with the PatchControl384 v1.5.2 software (Nanion) and the Biomek v1.0 interface (Beckman Coulter). Whole-cell recordings were conducted according to the recommended procedures of Nanion. Prior to recordings, dissociated cells were shaken at 200 RPM in a cell hotel reservoir at 10°C. After initiating the experiment, cell catch, seal and whole-cell formation, liquid application, recording, and data acquisition were all performed sequentially and automatically. For sodium channel recordings, the intracellular solution was (in mM) 10 CsCl, 110 CsF, 10 NaCl, 10 EGTA, and 10 HEPES (pH 7.2, osmolarity 280 mOsm), and the extracellular solution contained (in mM) 100 NaCl, 4 KCl, 40 NMDG, 2 CaCl_2_, 1 MgCl_2_, 5 glucose, and 10 HEPES (pH 7.4, osmolarity 298 mOsm). For sodium channel recordings, whole-cell experiments were performed at a holding potential (HP) of −100 mV at room temperature (RT, 20°C), and the sampling rate was set at 20 kHz. Peptides were diluted in the extracellular solution containing 0.3% bovine serum albumin. Na^+^ currents were triggered at a test potential of 0 or +10 mV for 50 ms or 1 s with a pulse every 5 s. The effects of each peptide on Na^+^ currents were measured at the end of a 10-min application time at RT (20°C). Activation curves were generated using 50 ms depolarizing steps ranging from −70 to +70 mV in 5-mV increments. Pulses were delivered at 5 s intervals. Steady-state inactivation curves were obtained by applying 1 s conditioning prepulses from −120 to 0 mV in 5-mV increments, followed by a 50 ms test pulse to 0 mV. Pulses were delivered every 10 s, during which cells were predominantly held at −100 mV prior to the prepulse and test pulse application. Quality controls (QCs) were imposed for the analysis of Na^+^ currents: QC1 imposed us to keep cells with seal resistances above 500 MΩ, whereas QC2 was meant to keep cells with current amplitudes above 400 pA and below 4 nA.

For human iPSC-derived cardiomyocytes recordings, cells were resuspended at 300 000 cells/mL in a solution composed of 50% culture media and 50% extracellular buffer containing (in mM) 140 NaCl, 4 KCl, 2 CaCl_2_, 1 MgCl_2_, 5 glucose, and 10 HEPES (pH 7.4, osmolarity 298 mOsm) supplemented with 5 µM nifedipine. Single-hole, low-resistance chips (3–5 MΩ) were used. The intracellular solution contained (in mM) 10 KCl, 110 KF, 10 NaCl, 1 MgCl_2_, 1 CaCl_2_, 10 Ethylene Glycol Tetra-acetic Acid (EGTA), and 10 HEPES (pH 7.2, osmolarity 280 mOsm). Currents were recorded and analysed as previously described for stable cell lines.

### Manual patch-clamp on HEK293-Na_v_1.5

Whole-cell Na_v_ currents were recorded at RT from HEK293 stably expressing hNa_v_1.5, using an Axopatch 200A amplifier (Axon Instruments, Molecular Devices). Voltage-clamp protocols were applied using the pClamp 10.4 software suite (Axon Instruments), interfaced with the electrophysiological setup through a Digidata 1440A digitizer (Axon Instruments). Patch-clamp pipettes were fabricated from borosilicate glass capillaries (outer diameter: 1.5 mm; inner diameter: 0.86 mm; Sutter Instrument) using a P-97 micropipette puller (Sutter Instrument), yielding a pipette resistance of 0.8–1.5 MΩ when filled with the internal solution. The internal solution contained (in mM) 10 CsCl, 110 CsF, 10 NaCl, 10 EGTA, and 10 HEPES (pH 7.2, osmolarity 280 mOsm). The external solution contained (in mM) 100 NaCl, 4 KCl, 40 NMDG, 2 CaCl_2_, 1 MgCl_2_, 5 glucose, and 10 HEPES (pH 7.4, osmolarity 298 mOsm). All chemicals were purchased from Sigma-Aldrich. After 80% series resistance compensation, 1000-ms depolarizing steps to −20 mV were applied from a HP of −120 mV to record sodium current under control conditions, following application of 10 nM AaH-II*. Sweeps were delivered every 5 s. Current signals were filtered at 10 kHz before digitization at 50 kHz and storage. Only cells with an access resistance <7 MΩ were included in the analysis, and input resistance was typically >1 GΩ. Leak currents were consistently <300 pA at the HP and were corrected offline. Cells displaying peak current amplitudes <500 pA or >10 000 pA were excluded from the analysis. Data were compiled and analysed using ClampFit 11.2 (Axon Instruments), Microsoft Excel, and GraphPad Software Prism.

### Manual patch-clamp on isolated adult rat cardiomyocytes

Whole-cell Na_v_ currents were recorded at RT from adult rat ventricular myocytes isolated as described for calcium imaging, using an Axopatch 200A amplifier (Axon Instruments, Molecular Devices) within 4 h of isolation. Voltage-clamp protocols were applied using the pClamp 10.4 software suite (Axon Instruments), interfaced with the electrophysiological setup through a Digidata 1440A digitizer (Axon Instruments). Patch-clamp pipettes were fabricated from borosilicate glass capillaries (outer diameter: 1.5 mm; inner diameter: 0.86 mm; Sutter Instrument) using a P-97 micropipette puller (Sutter Instrument), yielding a pipette resistance of 0.8–1.5 MΩ when filled with the internal solution. The internal solution contained (in mM) NaCl 5, CsF 115, CsCl 20, HEPES 10, and EGTA 10 (pH 7.35 adjusted with CsOH; ∼300 mOsm). The external solution contained (in mM) NaCl 10, CsCl 5, N-methyl-D-glucamine (NMDG) 104, TEA-Cl 25, HEPES 10, glucose 5, CaCl2 1, MgCl2 2, and CoCl2 2.5 (pH 7.4 adjusted with CsOH; ∼300 mOsm). All chemicals were purchased from Sigma-Aldrich. After 80% series resistance compensation, 1000-ms depolarizing steps to −20 mV were applied from a HP of −120 mV to record sodium current under control conditions, following application of 0.1 or 10 nM AaH-II*, and after subsequent addition of 100 nM or 10 µM TTX. Sweeps were delivered every 5 s. Current signals were filtered at 10 kHz before digitization at 50 kHz and storage. Only cells with an access resistance <7 MΩ were included in the analysis, and input resistance was typically >1 GΩ. Leak currents were consistently <300 pA at the HP and were corrected offline. Cells displaying peak current amplitudes <500 pA or >10 000 pA were excluded from the analysis. Data were compiled and analysed using ClampFit 11.2 (Axon Instruments), Microsoft Excel, and GraphPad Software Prism.

### AP recordings from human iPS-derived ventricular cardiomyocytes

The generation and recording of APs recorded by current clamp were performed on cardiomyocytes dissociated at D15.5 from right ventricular cardiac organoids after the onset of differentiation and maintained until D22.5 in 2D cultures in Petri dishes. Recordings were performed at 35–37°C in the perforated patch configuration using a VE-2 amplifier (Alembic Instruments, Montreal, QC, Canada) and Clampex 10.3 software (Molecular Devices, Sunnyvale, CA, USA). A 10 kHz low-pass filter is applied to the signal. The cardiomyocytes were perfused with a Tyrode’s solution containing 130 mM NaCl, 4 mM KCl, 1.2 mM NaH_2_PO_4_, 1.2 mM MgSO_4_(7H_2_O), 10 mM HEPES, 5 mM glucose, and 1 μM CaCl_2_ supplemented with 0.1 BSA (pH 7.4 with NaOH). Micropipettes, made from borosilicate capillaries and drawn in an automatic puller (Sutter Instrument, P-97), had 0.8–2.5 MΩ resistance and were filled with an internal solution containing 125 mM KGluconate, 20 mM KCl, 5 mM NaCl, 5 mM HEPES (pH 7.2 with KOH), and 65 μM of amphotericin B. APs were evoked at 1.43 Hz by using the dynamic clamp technique for injection of I_K1_ current^21,22^ to reach a resting potential of −90 mV. For drug administration, the perfused external solution was supplemented with 0.1 nM AaH-II* or 0.1 nM AaH-II* + 100 nM TTX. The analysis was performed using a programme written in R (RStudio, PBC, Boston, MA) by analyzing at least five consecutive action potentials in steady state.

### Calcium imaging in isolated rat cardiomyocytes

Eleven-week-old Wistar Han rats were euthanized by intraperitoneal (i.p.) injection of a lethal dose of pentobarbital (140 mg/kg, Euthasol®). Subsequently, the hearts were quickly excised and rinsed in cold calcium-free Tyrode solution with the following composition (in mM): 118 NaCl, 4.17 KCl, 1.2 MgSO_4_, 1.2 KH_2_PO_4_, 21 NaHCO_3_, 11 glucose, and 5 taurine, at pH 7.4. The aorta was promptly cannulated, and the hearts were perfused in a retrograde fashion with warm (37–38°C) Tyrode containing 1.25 mM CaCl_2_ to flush the coronary arteries of blood and then with a Tyrode solution supplemented with 1 mg/mL collagenase type 2 (Worthington Biochemical Corp, Lakewood, NJ) and 0.2 U.mL^−1^ protease type XIV (Sigma, Poole, UK) during 15–20 min. Once enzymatic digestion is completed, ventricles were shredded into small pieces with surgical scissors in 0.5 mM Ca^2+^ Tyrode solution, filtered through a 200-µm mesh, and then resuspended in 1 mM Ca^2+^ Tyrode solution for 1 h at RT. Isolated cardiomyocytes were incubated in the dark with Fura-2-AM calcium probe (2 µM in 20% v/v DMSO; Life Technologies) for 15 min. Cardiomyocytes were then loaded onto the IonOptix system (IonOptix Corporation, U.S.A.) and perfused with warm (37°C) Tyrode solution. Cardiomyocytes were stimulated at 0.5 Hz using a MyoPacer field stimulator (20 V, 7 ms) to achieve steady state. Fura-2 fluorescence (F) at 510 nm was used to measure cytoplasmic Ca^2+^ concentration variation by exciting with UV light at 340 and 380 nm. Cells were paced for 1 to 2 min, ensuring stabilization of the signals, and the effects of the peptides were measured at the end of a 5 min application time. 30-s-long periods were used as the unit duration (representing 15 Ca^2+^ transients) during control and after the perfusion of the compound to count the number of arrhythmogenic transients.

### *Ex vivo* cardiac electrophysiological recordings

Eleven-week-old Wistar-Han rats were first heparinized (heparin sodium, 0.5 U/g i.p.) and then euthanized by i.p. injection of 140 mg/kg pentobarbital (Euthasol®). Hearts were quickly excised and immersed in a cold modified Krebs-Henseleit solution (in mM): 116 NaCl, 5 KCl, 1.1 MgSO_4_, 7 H_2_O, 0.35 NaH_2_PO_4_, 27 NaHCO_3_, 10 glucose, and pH 7.4. The aorta was cannulated, and the hearts were perfused on a Langendorff system with warm (36.5–37.5°C) and oxygenated (95% O_2_/5% CO_2_) Krebs-Henseleit solution supplemented with 1.8 mM CaCl_2_, 1 mM lactate, and 0.2 mM pyruvate (control solution) at a constant flow rate of 12 mL/min. Once connected to the Langendorff perfusion system, hearts were perfused with control solution for 10 min to stabilize their activities. Electrical activity of the upper part of the right ventricle was assessed using a 64-electrode array (0.2-mm electrode diameter; 0.36-mm interelectrode distance) connected to an EMS64-USB-1002 amplifier. Single-lead ECG was acquired to monitor global cardiac electrical activity. Data were sampled at 10 kHz per channel and acquired by EMapRecord5 (Mappinglab, Oxford, UK). Data were obtained at baseline (2 min before compound perfusion) and during AaH-II* perfusion for 10 min at various concentrations. Data were analysed using EMapScope5 (Mappinglab, Oxford, UK). Activation times were determined as the point of maximal negative slope. All activation times were related to the timing of the first detected waveform and then used to draw activation maps. An average of three complexes was used for activation time and conduction velocity analyses, while interbeat interval was measured as the average of 10 consecutive intervals. The index of inhomogeneity was calculated as previously described.^23^ Arrhythmias were quantified over a period of 2 min in control and after peptide perfusion. Hearts exhibiting ventricular extrasystoles for more than 1 min were classified as arrhythmic.

### Electrocardiography

Eleven-week-old Wistar-Han rats were intubated using a custom-made 14G metallic cannula under general anaesthesia using sevoflurane (8% sevoflurane, FiO_2_ = 0.21, 2 L/min for induction and 4% sevoflurane, FiO_2_ = 0.21, 0.6 L/min for maintenance) to prevent respiratory depression associated with peptide administration. Rectal temperature was monitored continuously and maintained at 37–38°C using a heating pad. Six-lead ECGs were recorded with 25-gauge subcutaneous electrodes on a computer through an analogue-digital converter (IOX2 2.10.0.40, EMKA Technologies, France). The sampling rate was set to 1 kHz, and ECGs were recorded for 2 min as control and for 10 min after intravenous (i.v.) injection of 3, 15, or 30 µg/kg AaH-II* or 3, 9, 27, 81, or 243 µg/kg TTX using a 100 µL injection volume. Quantification of ECG parameters was performed in lead I (ECG Auto v3.5.5.28, EMKA Technologies), and data from at least 10 consecutive complexes were averaged. Standard criteria were used for interval measurements. The end of the T wave was defined as the point at which the slow component returned to the isoelectric line. The area of the T wave was measured between the end of the S wave and the end of the T wave. QT intervals were corrected for heart rate using the formula, QTc = QT/(RR/100)^1/2^.^24^ Blood samples (1 mL) were collected and centrifuged to extract plasma aliquots (50 µL minimal volume). Samples were stored at −20°C to later determine circulating peptide concentration by liquid chromatography-tandem mass spectrometry (LC-MS/MS).

### Liquid chromatography-tandem mass spectrometry

Circulating AaH-II* and TTX concentrations were analysed in plasma samples by LC-MS/MS. All solvents were LC-MS grade and purchased from Biosolve (Valkenswaard, Netherlands). Standard solutions of AaH-II* or TTX were prepared and serially diluted in water to obtain 10× standard solutions ranging 1–100 nmol/L. Standard solutions (10 µL) were then added to free plasma (90 µL) to get final concentrations ranging from 0.1 to 10 nmol/L. Standard and test samples were then deproteinized with 250 μL of acetonitrile. After centrifugation (10 min; 10 000× g; 4°C), supernatants were dried under a gentle stream of nitrogen for 30 min at RT. Dried samples were reconstituted in 100 µL of ultrapure water and transferred to vials for LC-MS/MS analyses. LC-MS/MS analyses were performed on a Xevo® TQD Absolute mass spectrometer with an electrospray interface and an Acquity PREMIER® UPLC^TM^ device (Waters Corporation, Milford, MA, USA). Samples (10 μL) were injected onto a CSH-PREMIER column (1.7 μm; 2.1 × 100 mm, Waters Corporation) held at 60°C, and AaH-II* or TTX was separated with a linear gradient of mobile phase B (acetonitrile, 0.1% formic acid) in mobile phase A (water, 0.1% formic acid) at a flow rate of 350 μL/min. Mobile phase B was kept constant for 0.5 min at 1%, linearly increased from 1 to 95% for 3 min, kept constant for 0.5 min, returned to the initial condition over 1 min, and kept constant for 1 min before the next injection. Targeted compounds were then detected by the mass spectrometer with the electrospray interface operating in the positive ion mode (capillary voltage, 3 kV; desolvation gas (N_2_) flow and temperature, 800 L/h and 450°C; source temperature, 120°C). The multiple reaction monitoring mode was applied for MS/MS detection: *m/z* 1203.6 → 1328.3 (cone: 30 V, collision: 30 eV) and *m/z* 1444.2 → 1804.7 (cone: 30 V, collision: 20 eV). Chromatographic peak areas constituted the detector responses. Standard solutions were used to plot calibration curves for quantification (linear regression, 1/× weighting, origin excluded).

### Statistics analyses

Values are represented as mean ± SEM (standard error of the mean). We performed all statistical analyses using GraphPad Prism 8.4.2. Comparisons between the control group and compound groups were performed using the Wilcoxon rank-sum test. For repeated measures, one-way ANOVA was performed to compare multiple groups among various conditions, followed by a Tukey post-test. A *P*-value lower than 0.05 was considered statistically significant.

## Results

### AaH-II* preferentially activates TTX-S cardiac Na_v_ channels

**Figure 1:**
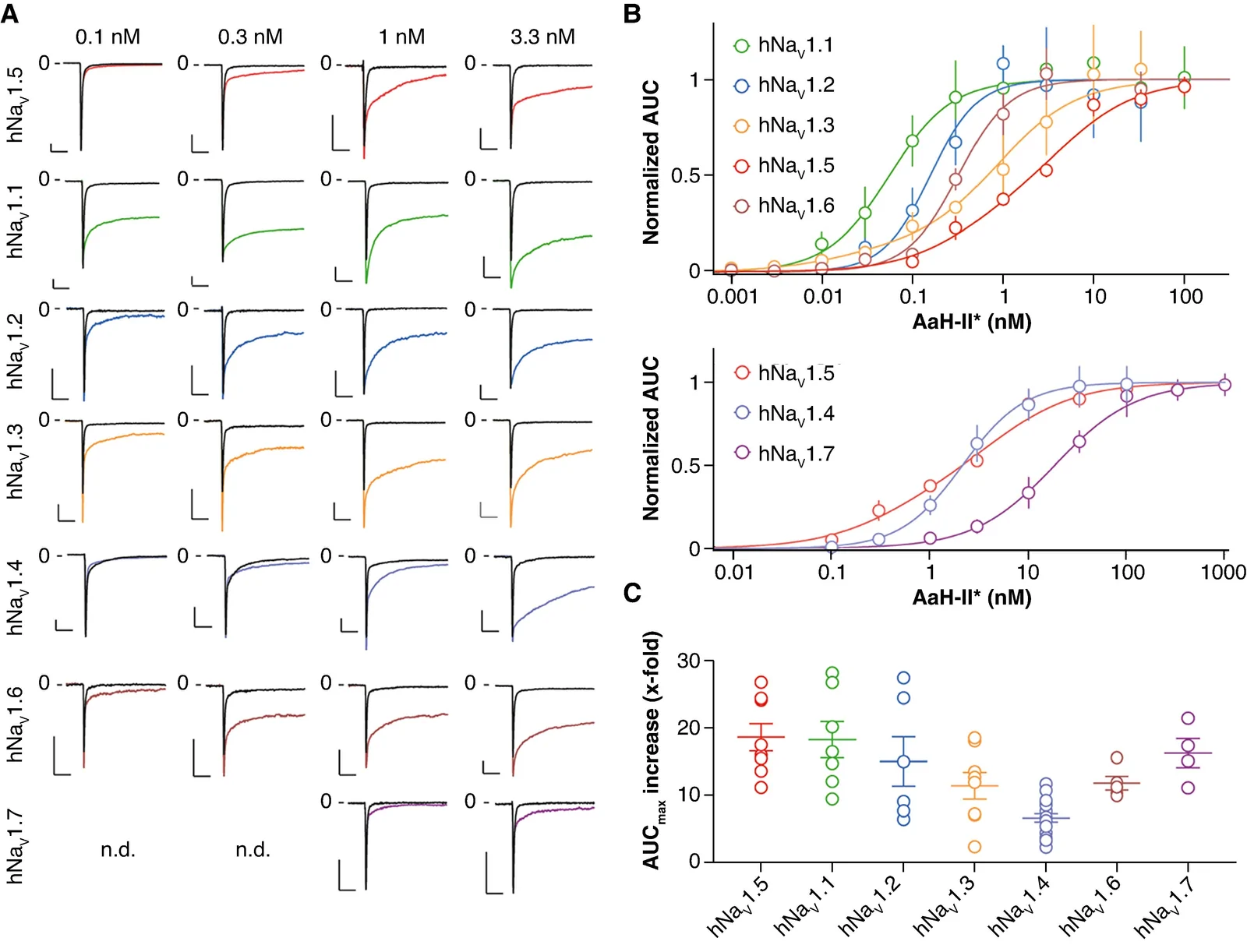
AaH-II* sensitivity of hNa_v_ channels. *A*, Representative current traces elicited at a test potential of 0 mV for various hNa_v_ channels stably expressed in HEK293 cells (all except hNa_v_1.7) or CHO cells (hNa_v_1.7) before and after application of 0.1, 0.3, 1, or 3.3 nM AaH-II*. n.d.: not determined. Scale bars: all 0.5 nA and 10 ms. The appearance of I_NaL_ also results in a peak current increase, as the activation process is no longer hidden by fast inactivation. *B*, Normalized average dose-response curves assessing the potency of AaH-II* on various hNa_v_ isoforms. IC_50_ values were calculated from single (hNa_v_1.1, hNa_v_1.2, hNa_v_1.4, hNa_v_1.6, and hNa_v_1.7) or double (hNa_v_1.3 and hNa_v_1.5) sigmoid fits of the data and provided in *Table 1*. *C*, Maximal AUC, comprising both peak and I_NaL_ increases, measured at the highest effective AaH-II* concentration for each hNa_v_ isoform. Mean ± SEM values are shown.

AaH-II was discovered before the cloning of the various Na_v_ isoforms was completed.^25^ Hence, it was mainly used as a radioactive tracer to characterize binding sites in various tissues and examine functional effects at neuromuscular junctions,^26^ but surprisingly, a complete pharmacological characterization of various human (h) Na_v_ isoforms has never been performed according to a standardized and statistically meaningful protocol. We took advantage of an automated high-throughput patch-clamp setup to perform this characterization with AaH-II* on a large set of hNa_v_ isoforms (*Figure 1*). The quality controls that we engaged for the analyses of the data ensure that the results should be of comparable quality to those performed by manual patch-clamp.^27–31^ Complete dose-response curves were constructed for hNa_v_1.5 on the basis of the area under the curve (AUC, reflecting the current integrals during depolarization) and compared to the dose-response curves for all TTX-S Na_v_ isoforms (hNa_v_1.1, hNa_v_1.2, hNa_v_1.3, hNa_v_1.4, hNa_v_1.6, and hNa_v_1.7). As shown, 0.1 nM AaH-II* is sufficient to trigger important I_NaL_ by hNa_v_1.1, hNa_v_1.2, hNa_v_1.3, and hNa_v_1.6, a concentration at which hNa_v_1.5 is largely spared (*Figure 1A*). Because inactivation is slowed, the activation process better reaches completeness, which explains the concomitant increase in peak currents that is occasionally observed along the development of I_NaL_. These TTX-S hNa_v_ isoforms thus have greater sensitivity to low concentrations of this peptide than the major cardiac Na_v_ isoform. A higher concentration of 0.3 nM is required to detect a noticeable I_NaL_ mediated by hNa_v_1.5 and hNa_v_1.4, while hNa_v_1.7 remains spared. A detectable I_NaL_ was measurable for hNa_v_1.7 only at 1 nM. Complete dose-response curves were built for all hNa_v_ isoforms and compared to the dose-response curve of hNa_v_1.5 (*Figure 1B*). hNa_v_1.1, hNa_v_1.2, hNa_v_1.3, and hNa_v_1.6 were all confirmed as being more sensitive to low AaH-II* concentrations than the hNa_v_1.5 isoform, suggesting that I_NaL_ from these TTX-S isoforms can be activated in isolation if these isoforms are present in cardiac tissue. In contrast, hNa_v_1.4 had equal sensitivity to AaH-II* as hNa_v_1.5, while hNa_v_1.7 was less sensitive, indicating that moderate concentrations of AaH-II* (<1 to 3 nM), susceptible to mildly activate hNa_v_1.5, should spare hNa_v_1.7. It is noticeable that the concentration-response curves for hNa_v_1.3 and hNa_v_1.5 had to be fitted by a double sigmoid, which would be coherent with the existence of two binding sites remaining for AaH-II*. Such a finding is not incoherent with the observation of two binding sites on the CryoEM structure of the Na_v_1.7-Na_v_PaS chimera.^15^ The EC_50_ values that quantify the AaH-II* dose required to half-maximally stimulate I_NaL_ are given in *Table 1*. Clearly, hNa_v_1.1, with an EC_50_ as low as 0.06 nM, was by far the most sensitive isoform to AaH-II*. The following rank order of sensitivity to AaH-II* was thus observed: hNa_v_1.1 > hNa_v_1.2 > hNa_v_1.6 > hNa_v_1.3 >> hNa_v_1.5 = hNa_v_1.4 >> hNa_v_1.7. *Figure 1C* also quantifies the maximum reachable level of I_NaL_ by AaH-II*. As seen, maximally effective AaH-II* concentrations provide the greatest maximal I_NaL_ stimulations for hNa_v_1.1 and hNa_v_1.5 (∼19-fold), while producing the lowest I_NaL_ stimulation for hNa_v_1.4 (∼six-fold).

**Table 1:** Comparison of AaH-II* and ATX-II selectivity among hNa_v_ subtypes

	AaH-II*				ATX-II			
	Mean EC_50_ (nM)	CI_95_ (nM)	*n*	vs. hNa_v_1.5	Mean EC_50_ (nM)	CI_95_ (nM)	*n*	vs. hNa_v_1.5
hNa_v_1.5	1.98	1.54–2.57	42	1	0.57	0.39–0.82	90	1
hNa_v_1.1	0.06	0.01–0.13	34	0.03	8.06	4.36–11.43	62	14.14
hNa_v_1.2	0.16	0.09–0.26	68	0.08	3.07	2.17–4.35	63	5.39
hNa_v_1.3	0.56	0.26–1.02	57	0.28	12.27	5.66–38.55	41	21.53
hNa_v_1.4	2.13	1.59–2.90	210	1.07	6.0	3.43–10.33	74	10.52
hNa_v_1.6	0.32	0.24–0.44	57	0.16	>100	—	36	>175.4
hNa_v_1.7	18.4	13.65–24.95	52	9.26	>100	—	31	>175.4

Ratio of selectivity is calculated as hNa_V_1.x/hNa_V_1.5.

CI_95_, 95% confidence interval (*n* cells).

**Figure 2:**
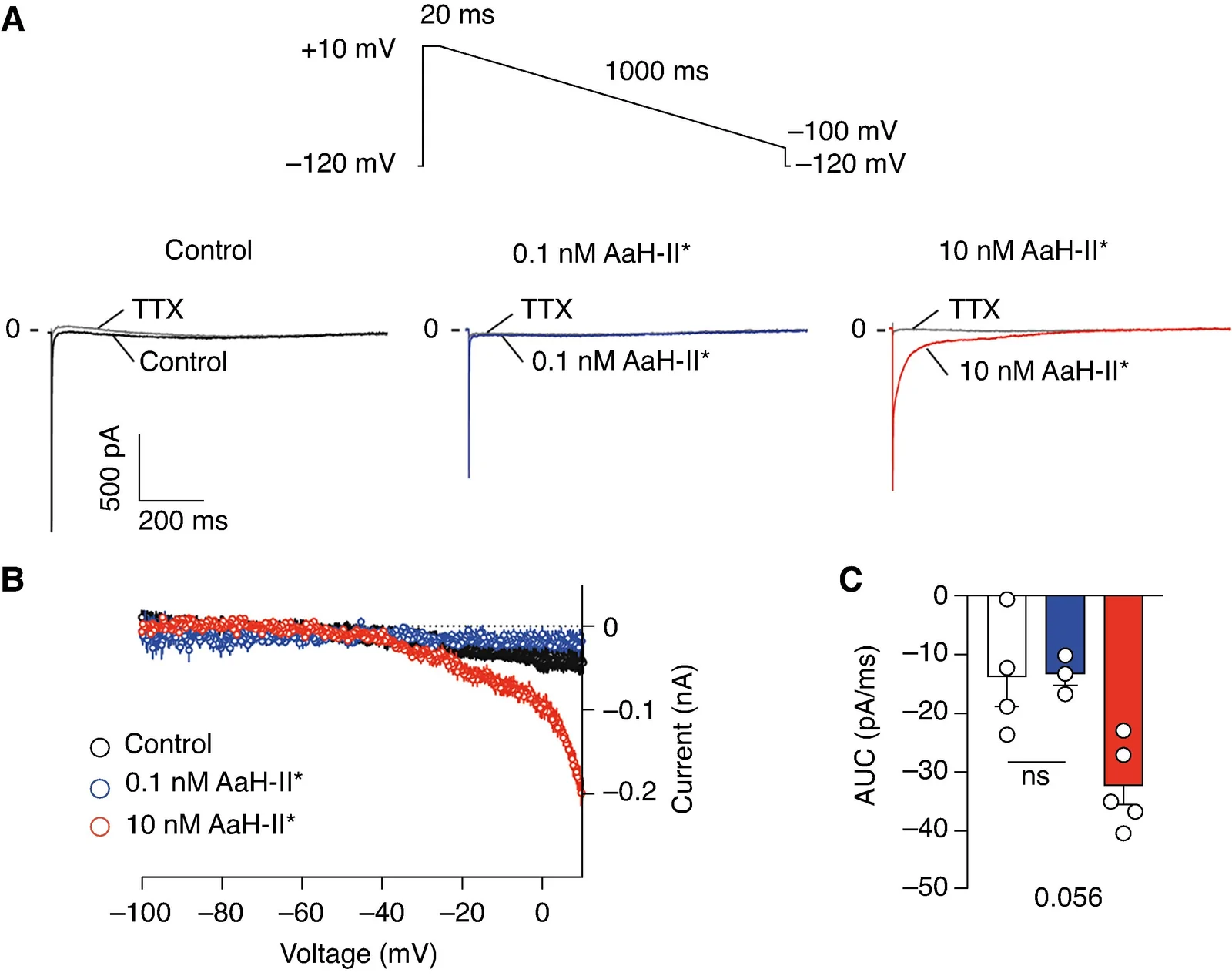
Lack of stimulated Na^+^ entry through hNa_v_1.5 at a low AaH-II* concentration. *A*, Top panel, voltage protocol used to simulate an action potential. Lower panels, actual representative Na^+^ currents through hNa_v_1.5 channels in control condition, 0.1 nM AaH-II* or 10 nM AaH-II* before and after application of 10 μM TTX. *B*, Persistent inward current traces during the descending ramp after subtraction of the current before by the current after 10 μM TTX for all three conditions. *C*, Quantification of the subtracted current density in all three conditions.

We also investigated how a maximally effective concentration of AaH-II* (10 nM) affects all other biophysical properties of Na_v_1.5 and TTX-S Na_v_ channels, including upon longer 1 s depolarization steps (see Supplementary material online, *Figures S2*–*S8*, Supplementary material online, *Table S1*). Interestingly, we found that hNa_v_1.5 differs from several TTX-S Na_v_ channels by the absence of AaH-II*-mediated I_NaL_ after 1 s depolarization (see Supplementary material online, *Figure S6* by automated patch-clamp and *Figure S9* by manual patch-clamp) and the lack of shift in the voltage-dependence of activation and inactivation (see Supplementary material online, *Figure S6*). hNa_v_1.4 resembled hNa_v_1.5 the most by the lack of effects of AaH-II* on those two parameters (see Supplementary material online, *Figure S5*). In contrast, several TTX-S Na_v_ channels displayed both a large AaH-II*-triggered hyperpolarizing shift in voltage-dependence of activation and a maintained I_NaL_ after 1 s pulse: hNa_v_1.1 (see Supplementary material online, *Figure S2*), hNa_v_1.3 (see Supplementary material online, *Figure S4*), and hNa_v_1.6 (see Supplementary material online, *Figure S7*). hNa_v_1.7 presented the AaH-II*-mediated shift in voltage-dependence of activation but lacked sufficient I_NaL_ at 1 s depolarization, most likely because of incomplete stimulation of the channel at 10 nM (see Supplementary material online, *Figure S8*). hNa_v_1.2 displayed a significant I_NaL_ current at 1 s depolarization but lacked a significant shift in voltage-dependence of activation (see Supplementary material online, *Figure S3*). Altogether, these data suggest that, at low concentrations, AaH-II* is an excellent selective promoter of the activity of several TTX-S hNa_v_ isoforms by inducing and maintaining I_NaL_ during long depolarizing steps, while also triggering channel openings at lower voltages. Our data strongly suggest that using 0.1 nM AaH-II* should selectively activate several TTX-S hNa_v_ channels while sparing hNa_v_1.5. To ensure that this statement is correct, we examined the capacity of 0.1 nM AaH-II* to generate Na^+^ entry through hNa_v_1.5 channels in an action potential-mimicking protocol (*Figure 2*). As can be seen, only 10 nM AaH-II* is capable of generating a measurable Na^+^ current in these conditions, while 0.1 nM AaH-II* was indistinguishable from the control condition. These data leave us confident that 0.1 nM AaH-II* is fully selective for several TTX-S Na_v_ channels.

For the purpose of comparison, we also examined the ATX-II sensitivity of these hNa_v_ isoforms in the same standardized conditions as those used for AaH-II* (see Supplementary material online, *Figure S10*). As shown, 0.1 to 0.3 nM ATX-II induces a significant I_NaL_ for hNa_v_1.5 that is not detected for TTX-S hNa_v_ isoforms, except maybe marginally on hNa_v_1.2. According to the ATX-II dose-response curves performed, the EC_50_ value for inducing hNa_v_1.5 I_NaL_ was 0.57 nM (*Table 1*). The rank order of sensitivity to ATX-II was as follows: hNa_v_1.5 > hNa_v_1.2 > hNa_v_1.4 ≥ hNa_v_1.1 ≥ hNa_v_1.3 >>> hNa_v_1.6 = hNa_v_1.7. We found no evidence that ATX-II affects hNa_v_1.6 or hNa_v_1.7 at concentrations up to 33 nM. For hNa_v_1.6, this result is a noticeable difference compared to AaH-II* (*Figure 1* and Supplementary material online, *Figure S7*). In addition, the maximal reachable I_NaL_ was significantly lower for hNa_v_1.3 and hNa_v_1.4 compared to hNa_v_1.1 and hNa_v_1.2 (see Supplementary material online, *Figure S10*). The rank order of sensitivity and the lack of sensitivity of hNa_v_1.6 and hNa_v_1.7 to ATX-II are therefore inverse to those observed for AaH-II*, indicating that ATX-II can be used for preferential activation of Na_v_1.5 I_NaL_ in cardiac tissues at 0.1 to 0.3 nM, while AaH-II* is an indicated pharmacological tool for the preferential activation of several TTX-S I_NaL_ at 0.1 nM.

### AaH-II* pharmacological effect does not preclude TTX sensitivity

Because TTX will be used to assess the importance of TTX-S I_NaL_ in cardiac arrhythmias, we made sure that AaH-II*-modified currents were still sensitive to 100 nM TTX. As shown, addition of 10 nM AaH-II* to hNa_v_1.6-expressing cells induces an important uprise in I_NaL_ (see Supplementary material online, *Figure S11A*–*S11C*). A further addition of 100 nM TTX leads to a complete block of hNa_v_1.6 currents, which is coherent with totally different Na_v_ binding sites and modes of action of these pharmacological agents (pore block for TTX and gating modifier for AaH-II*).^15,32–34^ The opposite experiment was also performed wherein hNa_v_1.6 currents were first fully blocked by 100 nM TTX, and the subsequent application of 10 nM AaH-II* failed to activate any form of current, including I_NaL_ (see Supplementary material online, *Figure S11D*–*S11F*).

### Low AaH-II* concentrations also trigger I_NaL_ in human and adult rat cardiomyocytes through TTX-S Na_v_ channels

**Figure 3:**
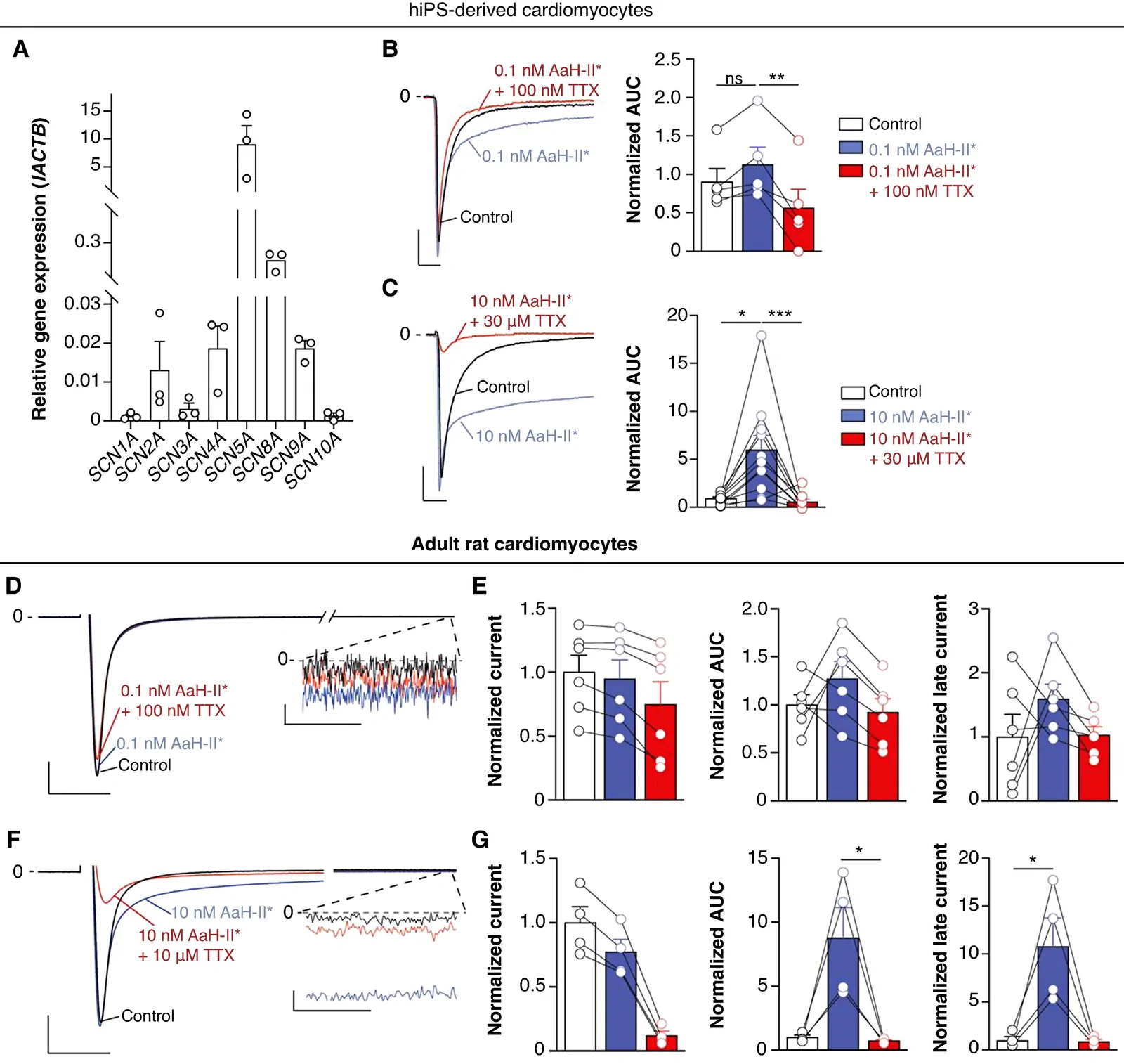
AaH-II* stimulates TTX-S Na_v_ channels in human iPS-derived and rat ventricular cardiomyocytes. *A*, qRT-PCR experiments quantifying the relative gene expression of seven hNa_v_ channels in right ventricle cardiomyocytes derived from iPS cells. *B*, Left panel: representative Na^+^ currents from human iPS-derived cardiomyocytes before and after application of 0.1 nM AaH-II* (first) and 100 nM TTX + 0.1 nM AaH-II* (second). Scale bars: 5 ms/1 nA. Right panel: average normalized AUC illustrating I_NaL_ increase by AaH-II* and decrease by 100 nM TTX from TTX-S Na_v_ channels. *C*, as in *B* but using 10 nM AaH-II* that produces I_NaL_ from both TTX-S and Na_v_1.5 channels. 30 μM TTX inhibits all Na_v_ isoforms. Scale bars: 5 ms/1 nA. *, *P* < 0.05; **, *P* < 0.01; ***, *P* < 0.001. *D*, Representative Na^+^ currents from adult rat cardiomyocytes (holding potential −120 mV, test potential −20 mV) before and after application of 0.1 nM AaH-II* (first) and 100 nM TTX + 0.1 nM AaH-II* (second). Scale bars: 200 ms/2 nA and inset 5 ms/2 pA. *E*, Quantification of all parameters: peak current amplitude, AUC, and I_NaL_ on *n* = 6 cardiomyocytes. *F, G*, Same as *D* and *E*, but using 10 nM AaH-II* to act on the entire Na_v_ population and 10 μM TTX to block all Na_v_ channels, including the TTX-resistant Na_v_1.5 isoform. Scale bars: 200 ms/2 nA and inset 5 ms/20 pA.

We next investigated whether a low AaH-II* concentration also activates I_NaL_ through TTX-S Na_v_ channels in human iPS-derived cardiomyocytes (*Figure 3A–3C*). We first examined the identity of hNa_v_ channels expressed in human right ventricle cardiomyocytes derived from iPS cells by qRT-PCR experiments (*Figure 3A*). As expected, hNa_v_1.5 is by far the main Na_v_ channel expressed in cardiomyocytes, followed by hNa_v_1.6 > hNa_v_1.4 > hNa_v_1.2 >> hNa_v_1.3 > hNa_v_1.8 = hNa_v_1.1. As a whole, these TTX-S Na_v_ channels represent less than 5% of the total Na_v_ channel population expressed. The identification of hNa_v_1.6 as the predominant TTX-sensitive Na_v_ isoform is relevant to our earlier findings, making this channel type particularly sensitive to 0.1 nM AaH-II* (maintained I_NaL_ at 1 s depolarization and a negative shift in the voltage-dependence of activation) (*Figure 1* and Supplementary material online, *Figure S7*). We next tested 0.1 nM AaH-II* on the Na^+^ current carried by these human cardiomyocytes to determine if selective activation of TTX-S Na_v_ channels could lead to I_NaL_ in spite of the lower representation of these channels. Application of 0.1 nM AaH-II* triggered a characteristic I_NaL_ that could be entirely blocked by 100 nM TTX, indicating the factual contribution of TTX-S Na_v_ channels to this current (*Figure 3B*). The most likely contributors to AaH-II*-mediated I_NaL_ are hNa_v_1.6 and hNa_v_1.2, since hNa_v_1.4 was barely sensitive to 0.1 nM AaH-II*. A minor contribution of hNa_v_1.3 to this I_NaL_ current also remains a possibility. With higher concentrations of AaH-II* (10 nM), acting on both TTX-S Na_v_ channels and Na_v_1.5, a greater I_NaL_ could be recorded, which was fully blocked by 30 μM TTX (*Figure 3C*). These findings confirm that human cardiomyocytes contain a small proportion of TTX-S Na_v_ channels that selectively induce I_NaL_ at low AaH-II* concentrations.

Next, we investigated the situation in adult rat ventricular cardiomyocytes using manual patch-clamp (*Figure 3D–3G*). Total Na_v_ Na^+^ currents were recorded by 1-s test pulses at −20 mV from a holding potential of −120 mV. Mitigated effects were observed at 0.1 nM AaH-II*. None of the cells witnessed an increase in peak current amplitude, but half of the cells responded with an increased AUC and I_NaL_ at 1 s depolarization (*Figure 3D* and *3E*). These results indicate heterogeneity in Na_v_ channel composition from individual cardiomyocytes. In contrast, 10 nM AaH-II* triggered both a significant increase in AUC and I_NaL_ in all cells recorded (*Figure 3F* and *3G*), which is coherent with the predominant expression of Na_v_1.5 and indicates proper peptide application and pharmacological response. All these responses were blocked by 10 μM TTX, demonstrating the specificity of AaH-II* effects.

### I_NaL_ by TTX-S Na_v_ channels trigger action potential elongation in human iPS-derived cardiomyocytes

**Figure 4:**
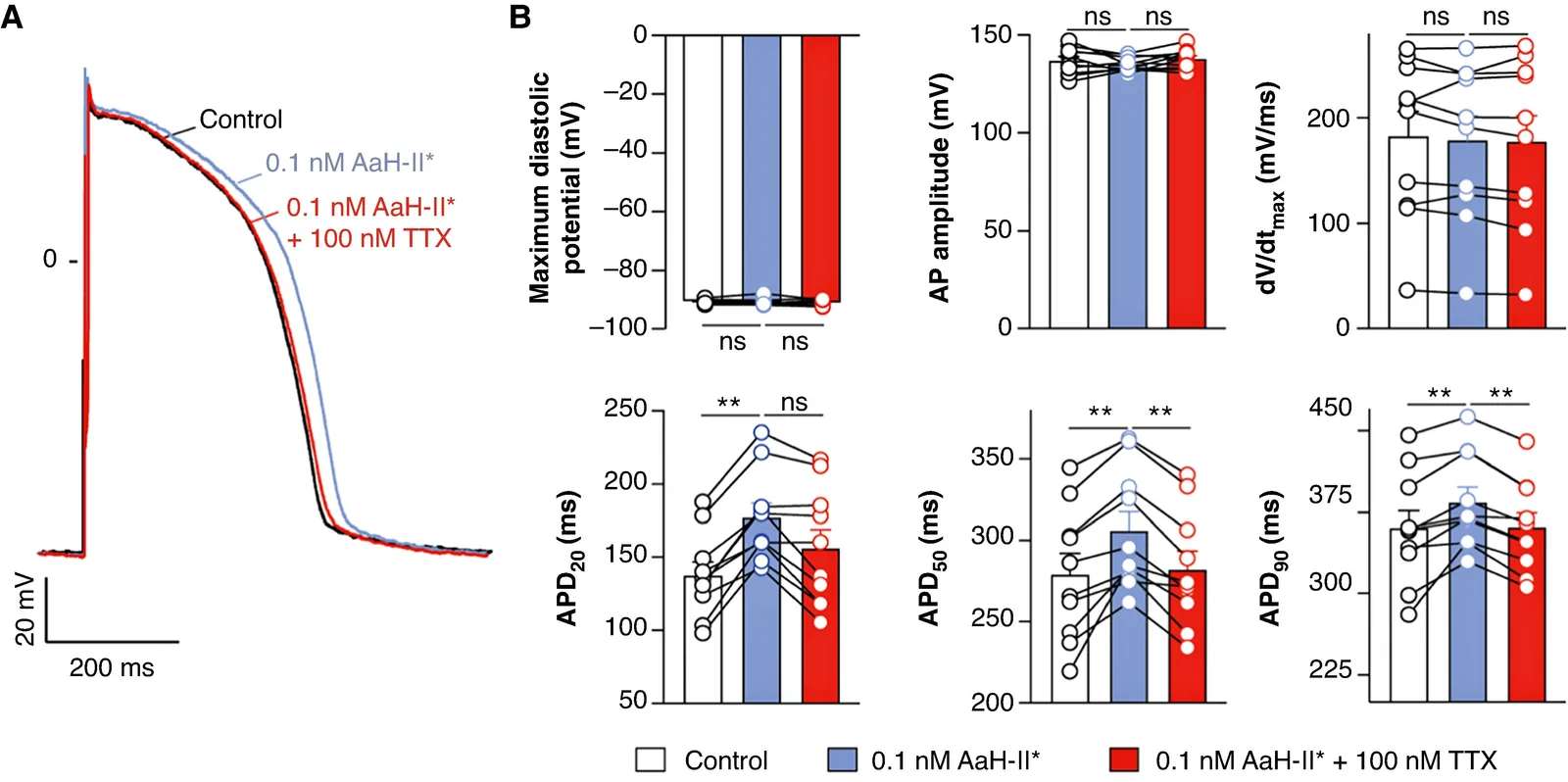
Selective activation of TTX-S Na_v_ channels by AaH-II* prolongs the APD of human iPS-derived cardiomyocytes. *A*, Representative APs of a right ventricle cardiomyocyte in control condition, after 0.1 nM AaH-II* application, and the further addition of 100 nM TTX to block TTX-S Na_v_ channels. *B*, Quantification of various AP parameters (*n* = 10). **, *P* < 0.01.

Since a significant I_NaL_ could be triggered through TTX-S Na_v_ channels by 0.1 AaH-II* in human cardiomyocytes, it is expected that the same concentration should affect the properties of their APs. The AP recordings performed in dynamic current clamp illustrate that the main effect of 0.1 nM AaH-II* is to increase the AP duration (APD) APD_20_, APD_50_, and APD_90_ (*Figure 4A* and *4B*). As expected for a peptide that mainly slows Na_v_ inactivation kinetics, the maximal diastolic potential, the AP amplitude, and the dV/dt_max_ were not significantly affected by 0.1 nM AaH-II* (*Figure 4B*).

### Dose-dependent proarrhythmic cellular phenotype induction by AaH-II* and TTX sensitivity of the response

**Figure 5:**
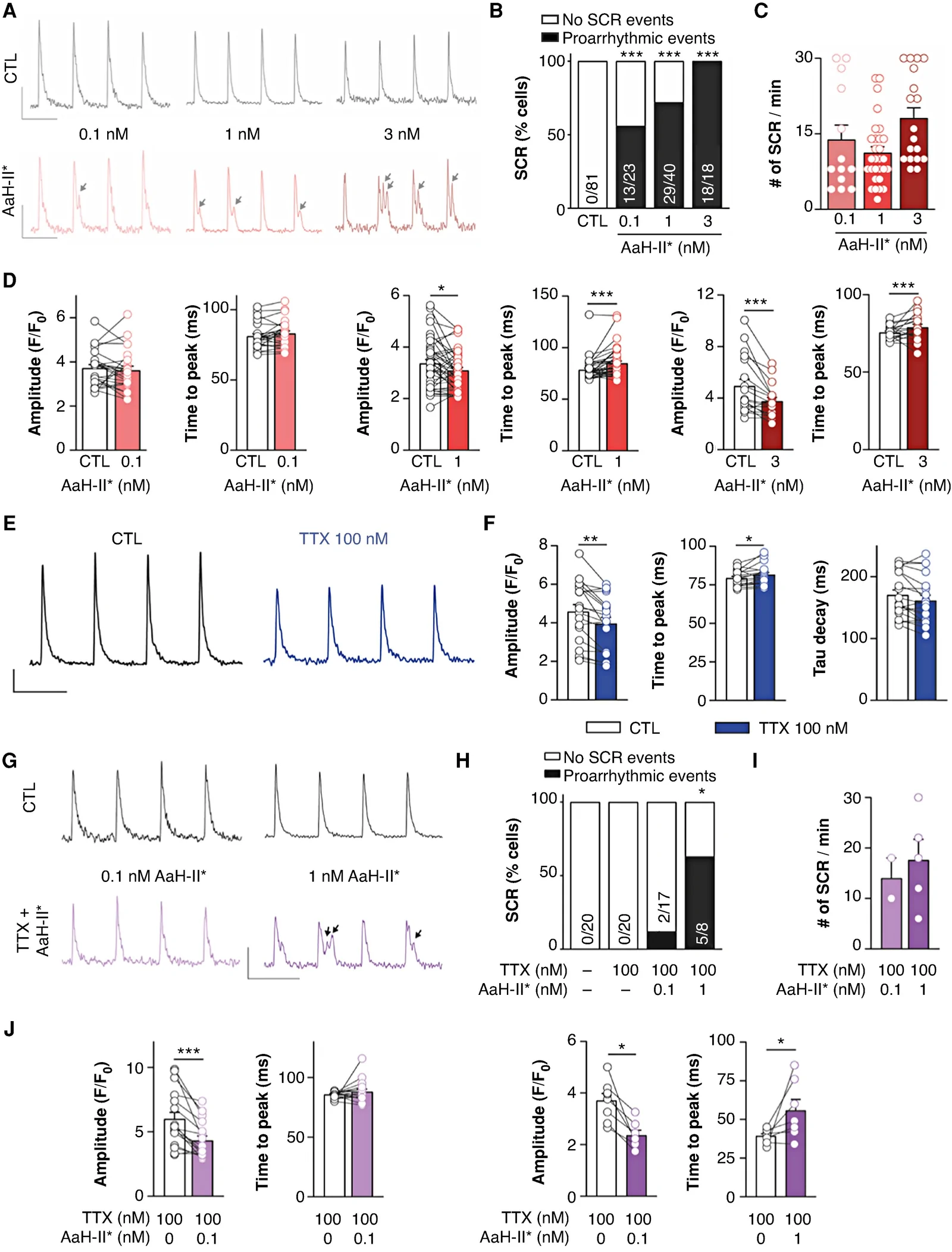
Effect of AaH-II* on calcium release from adult rat ventricular cardiomyocytes. *A*, Calcium transients in control condition and after addition of 0.1, 1, or 3 nM AaH-II*. SCR events are shown by arrows. Scale bars: 1 F/F_0_; 2 s. *B*, Percentage of cells presenting SCR events in various AaH-II* concentration conditions. *C*, Number of SCR events per min of recording as a function of AaH-II* concentration. *D*, Quantification of the properties of elicited calcium transients in control condition and after application of 0.1, 1, or 3 nM AaH-II*. *E*, Calcium transients in control condition and after perfusion of 100 nM TTX. Scale bars: 1 F/F_0_, 2 s. *F*, Quantification of amplitude, time to peak, and tau decay of calcium transients in control condition and after perfusion of 100 nM TTX. *G*, Representative calcium transients recorded in control condition and after application of 100 nM TTX. Scale bars: 1 F/F_0_; 2 s. *H*, Percentage of cells displaying SCR in various pharmacological conditions. *I*, Number of SCR per min for cells that displayed SCR. *J*, Properties of the calcium transients in the double pharmacological treatment condition (100 nM TTX + 0.1 nM AaH-II*).

Freshly dissociated ventricular cardiomyocytes from adult rats were electrically stimulated at 0.5 Hz to investigate the shape of spontaneous calcium release (SCR) events with the IonOptix system in various experimental conditions. We first confirmed the proarrhythmic cellular effects of ATX-II. At 0.1 nM, it should induce I_NaL_ selectively from the Na_v_1.5 isoform. In these conditions, the peptide triggers the appearance of proarrhythmic SCR events in 3 out of 11 cells and with a frequency above 15 events per min (see Supplementary material online, *Figure S12A*–*S12C*). In addition, the amplitudes of the calcium transients are reduced, while their time to peak is increased (see Supplementary material online, *Figure S12D*). Both the percentage of cells displaying proarrhythmic SCR events and the frequency of these events increase with higher ATX-II concentrations (1 and 3 nM). These observations are coherent with the fact that higher ATX-II concentrations produce greater I_NaL_ from Na_v_1.5, with 3 nM ATX-II producing a sub-maximal effect (see Supplementary material online, *Figure S10*). However, 3 nM ATX-II is also a concentration at which several TTX-sensitive Na_v_ isoforms display I_NaL_ (hNa_v_1.1, hNa_v_1.2, hNa_v_1.3, and hNa_v_1.4), suggesting that their participation in the enhanced proarrhythmic phenotype of the cardiomyocytes cannot be ruled out. To test the potential involvement of TTX-S Na_v_ isoforms in a proarrhythmic phenotype of cardiomyocytes, we also tested the appearance of SCR events upon application of various concentrations of AaH-II*. At 0.1 nM AaH-II*, a concentration that should selectively trigger I_NaL_ from TTX-S Na_v_ channels while largely sparing Na_v_1.5, SCR events are also observed in 13 out of 23 cells and with an average frequency of 14 SCR/min (*Figure 5A–5C*). The ratio of responding cells is thus coherent with the ratio of cells producing I_NaL_ at 0.1 nM AaH-II* (*Figure 3E*). Increasing the AaH-II* concentration to 1 or 3 nM further enhances the probability of proarrhythmic events, most likely by enhancing the TTX-S I_NaL_ amplitude and simultaneously recruiting the Na_v_1.5 I_NaL_ component (*Figure 5A–5C*). These higher concentrations of AaH-II* slightly reduce the amplitude of the calcium transients and slow the time to peak, possibly through L-type Ca^2+^ channel inactivation and reduced Ca^2+^ entry (*Figure 5D*). This first set of data indicates that the selective activation of TTX-S Na_v_ channels does trigger a proarrhythmic phenotype in the absence of Na_v_1.5 contribution. A further demonstration of the involvement of TTX-S Na_v_ isoforms in a proarrhythmic phenotype can be achieved by blocking TTX-S Na_v_ channels with a mild TTX concentration (100 nM) that spares Na_v_1.5. As shown, 100 nM TTX does not by itself trigger the appearance of SCR events (*Figure 5E*). It does, however, reduce the amplitude and increase the time to peak of regular calcium transients moderately. These results are coherent with a tight coupling between TTX-S Na_v_ channels and the Ca^2+^ mobilization machinery. Surprisingly, the mild effect of 100 nM TTX on calcium transients (*Figure 5E*) is not mirrored by an increase in calcium transients by 0.1 nM AaH-II* (*Figure 5D*), suggesting that TTX-S Na_v_ isoforms are already maximally effective in triggering calcium transients in the absence of any stimulating drug. In the presence of 100 nM TTX, 0.1 nM AaH-II* triggers SCR events with a much lower frequency than in the absence of TTX, with two responding cells over 17 (*Figure 5G–5I*). These results confirm that I_NaL_ from TTX-S Na_v_ isoforms has the potential to trigger proarrhythmic cellular phenotypes. They are coherent with a minimal contribution of Na_v_1.5 to I_NaL_ at this AaH-II* concentration (*Figures 1* and *2*, Supplementary material online, *Figure S6*). Conversely, at 1 nM AaH-II*, a concentration that leads to the concomitant activation of TTX-sensitive and Na_v_1.5 I_NaL_, SCR events become far more frequent (*Figure 5G–5I*) in proportions that more closely resemble what is observed in the absence of 100 nM TTX (*Figure 5A–5C*). As observed for TTX alone, the condition TTX + AaH-II* (0.1 nM) leads to decreased calcium transient amplitude and time to peak (*Figure 5J*). Overall, these data demonstrate that I_NaL_ of cardiac TTX-S Na_v_ isoforms triggers proarrhythmic events *in vitro* in isolated rat cardiomyocytes.

### *Ex vivo* proarrhythmic effects of AaH-II* on TTX-S Na_v_ channels

**Figure 6:**
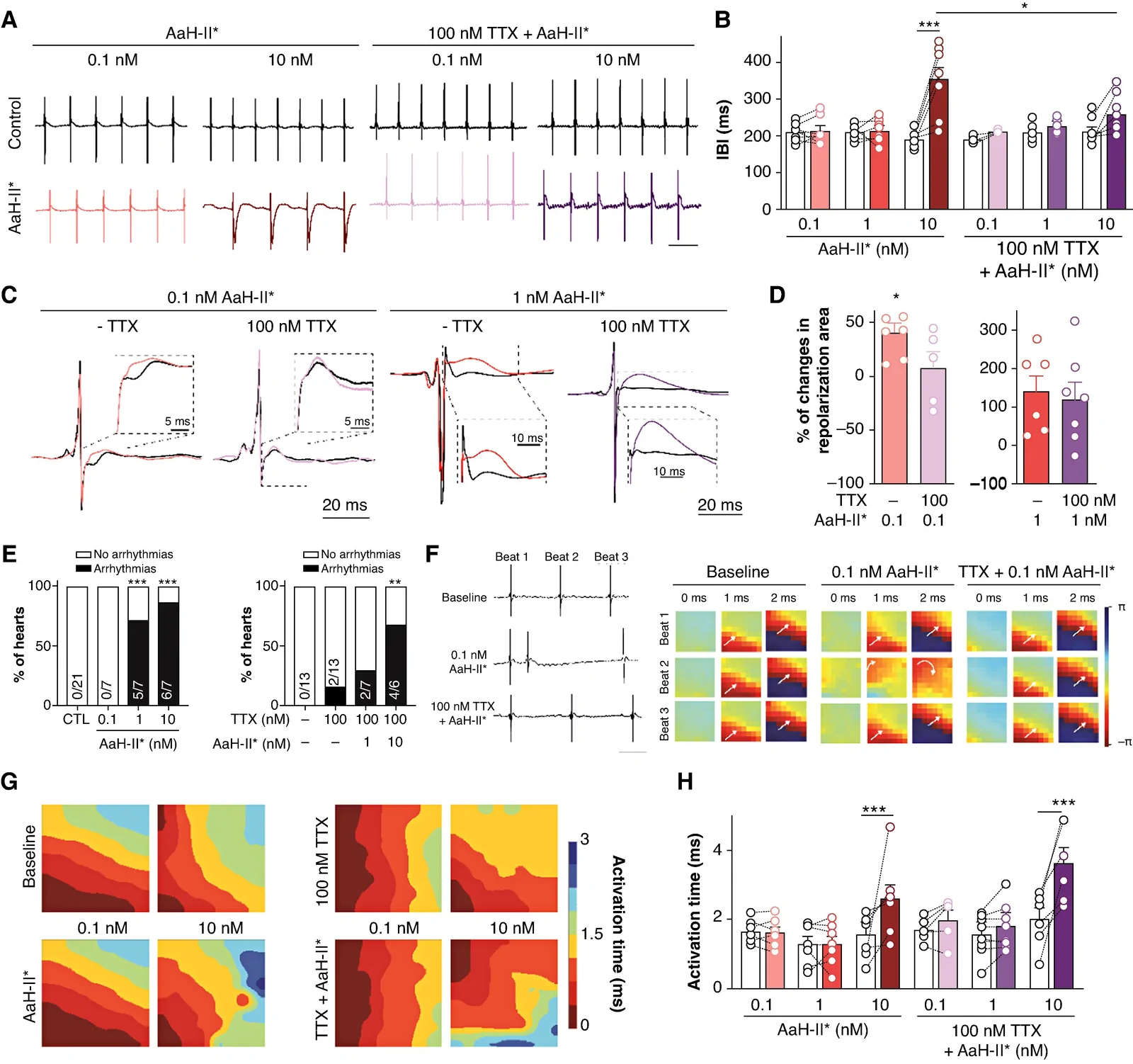
Arrhythmogenic effects of AaH-II* in isolated rat heart models. *A*, Representative MEA recordings in control conditions and after perfusion of AaH-II* at various concentrations without and with pre-treatment with 100 nM TTX. Scale bar: 250 ms. *B*, Quantification of ventricular interbeat intervals (IBI) in various pharmacological conditions. *, *P* < 0.05, ***, *P* < 0.001. *C*, Representative ventricular local field potentials at 0.1 and 1 nM AaH-II* without and with 100 nM TTX pre-treatment. *D*, Quantification of the percentage of increase in repolarization area in various pharmacological conditions. *E*, Quantification of arrhythmogenic hearts in various pharmacological conditions. *F*, Representative MEA recordings and phase maps highlighting the arrhythmogenic impact of AaH-II* and 100 nM TTX pre-treatment on the directionality of the electrical propagation. The white arrow indicates this directionality. Scale bar: 100 ms. *G*, Activation maps in various pharmacological conditions. *H*, Quantification of activation time in various pharmacological conditions.

To check the proarrhythmic consequences of AaH-II* on cardiac tissue, we first evaluated its effects in isolated rat heart models to prevent confounding effects due to the modulation of neuronal afferences in which TTX-S channels are expressed. As measured by multielectrode array (MEA) on the upper part of the right ventricle (located near or on the right ventricular outflow tract), 0.1 and 1 nM AaH-II* have no effect on the interbeat interval, whereas 10 nM AaH-II* induces significant ventricular bradycardia (1.9-fold rhythm reduction) and heterogeneous repolarization (*Figure 6A* and *6B*). A rhythm reduction of this magnitude is expected to induce a global conduction block at the cardiac level. A significant proportion of this bradycardia is prevented by pre-treatment with 100 nM TTX, indicating the involvement of TTX-S Na_v_ channels (*Figure 6A* and *6B*). The remaining bradycardia, which is still significant, appears to be due to the small bradycardic effect of TTX itself (see Supplementary material online, *Figure S13A* and *S13C*). A total of 100 nM TTX is the concentration accepted as selectively blocking TTX-S Na_v_ channels, without affecting Na_v_1.5.^35^ This is indeed validated by the observation that perfusion of 100 nM TTX does not affect ventricular conduction velocity supported by Na_v_1.5 (see Supplementary material online, *Figure S13B*).^36,37^ Therefore, we conclude that the bradycardic effect of 10 nM AaH-II* requires the participation of TTX-S Na_v_ channels, as previously suggested.^38^ At 10 nM AaH-II*, I_NaL_ should also be carried by Na_v_1.5 in cardiac tissue because this concentration is 100-fold higher than the concentration that largely spares Na_v_1.5 *in vitro*. These data are coherent with a previous report indicating that I_NaL_ supported by gain-of-function mutations of Na_v_1.5 is not bradycardic.^24^ A closer examination of the shape of the ventricular local field potential indicates that 0.1 nM AaH-II* significantly increases the repolarization area by 40.3 ± 8.0%. This effect is prevented by 100 nM TTX (*Figure 6C* and *6D*). This result validates the involvement of TTX-S I_NaL_ in the repolarization gradient of ventricular tissue. At 1 nM AaH-II*, the repolarization area is further increased to 140.6 ± 43.0%, an effect that is no longer prevented by 100 nM TTX (*Figure 6C* and *6D*). This latter result indicates that perfusion of 1 nM AaH-II* leads to an increase in AaH-II* tissue concentration above 0.1 nM that is sufficient to increase I_NaL_ carried by Na_v_1.5. Therefore, the cardiac tissue should witness a concentration of AaH-II* in the vicinity of Na_v_1.5 that is between 0.1 and 1 nM. The increase in the area of repolarization by 0.1 nM AaH-II* was insufficient to induce recurrent premature ventricular contractions (PVCs) in all seven hearts studied (*Figure 6E*). However, evident signs of arrhythmias were observed, as witnessed by the presence of a few PVCs and altered directionality of electrical propagation, indicating that the hearts were arrhythmic (*Figure 6F*). The further increase of the repolarization gradient at 1 nM AaH-II*, by the combined I_NaL_ activation from Na_v_1.5 and the augmented I_NaL_ carried by TTX-S Na_v_ channels, leads to the emergence of sustained arrhythmias in 5/7 hearts studied *ex vivo* (*Figure 6E*). Pre-treatment of the hearts with 100 nM TTX largely suppresses the initial signs of arrhythmias at 0.1 nM AaH-II* and restores the directionality of the electrical propagation (*Figure 6F*). The level of suppression of arrhythmias by TTX is almost complete if 1 nM AaH-II* is used, with part of these arrhythmias being triggered by TTX itself (*Figure 6E*). These results indicate that inhibition of TTX-S Na_v_ channels helps prevent arrhythmias triggered by the I_NaL_ resulting from TTX-S channels and the low proportion of Na_v_1.5 activated by AaH-II*. However, if a larger activation of I_NaL_ carried by Na_v_1.5 is promoted by 10 nM AaH-II*, then the arrhythmogenic phenotype is only slightly decreased by 100 nM TTX pre-treatment (from 6/7 arrhythmogenic hearts to 4/6; *Figure 6E*). We next examined the impact of AaH-II* on cardiac conduction in the right ventricle. As shown, both 0.1 and 1 nM AaH-II* do not affect conduction (*Figure 6G* and *6H* and Supplementary material online, *Figure S13*), suggesting that TTX-S channels are not involved in this process. It is also in agreement with the lack of effect of TTX on conduction velocity (see Supplementary material online, *Figure S14*). However, 10 nM AaH-II* significantly increases activation time (*Figure 6G* and *6H*), which is not prevented by 100 nM TTX, indicating that Na_v_1.5 is the major contributor to conduction velocity, as shown previously.^39^ We suppose that the cells excessively depolarize, leading to conduction velocity defects, as suggested in LQT3 models.^40,41^ In addition to slowing the conduction velocity, 10 nM AaH-II* increases the heterogeneity of conduction (see Supplementary material online, *Figure S13*).

### *In vivo* proarrhythmic effects of AaH-II*

**Figure 7:**
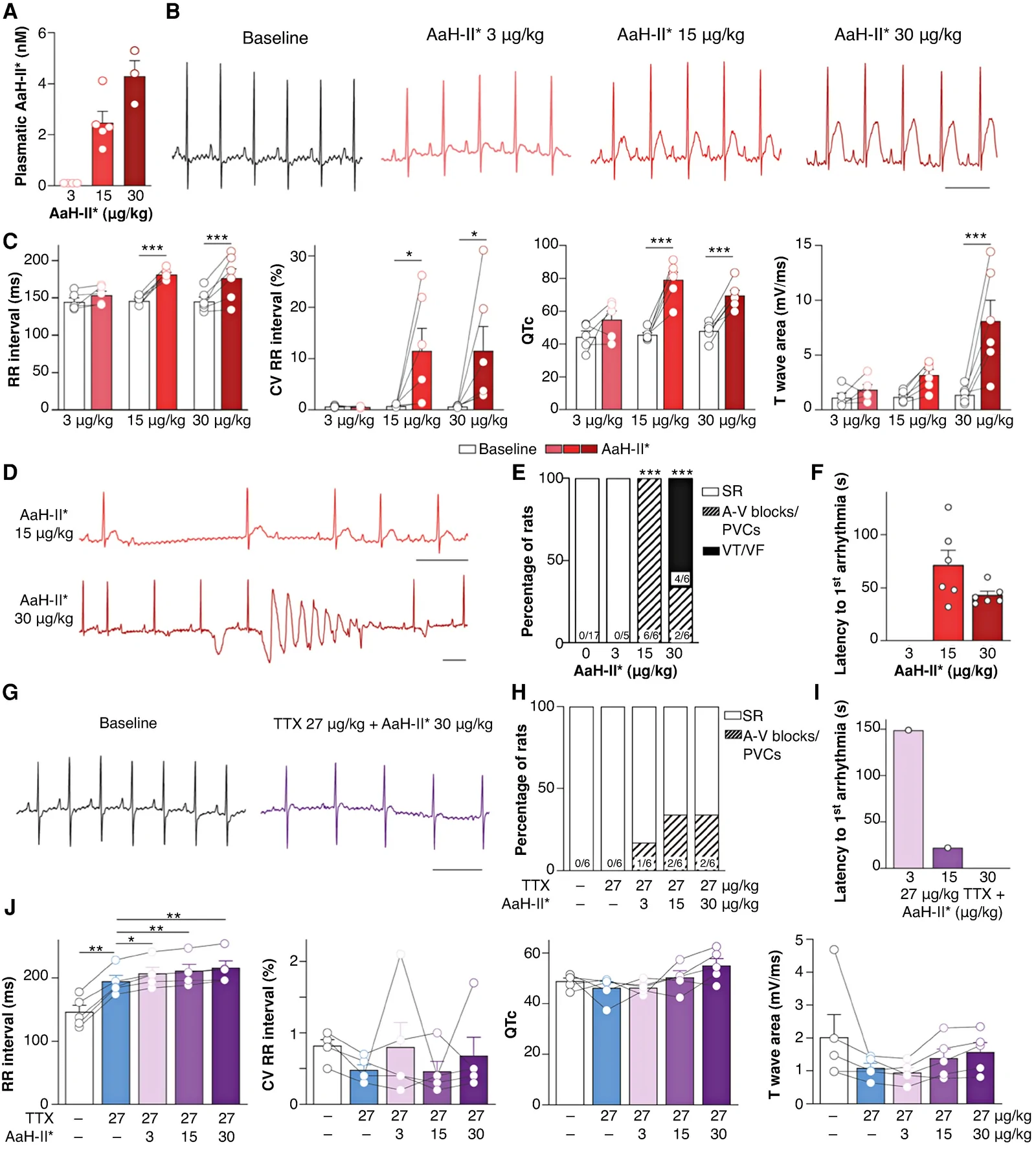
*In vivo* pro-arrhythmogenic effects of AaH-II*. *A*, Circulating plasmatic concentrations of AaH-II* at three injected doses in rats. *B*, Representative ECG recordings in control conditions or at three different doses of AaH-II*. Scale bar: 200 ms. *C*, Quantification of ECG parameters at the three doses of AaH-II*. *, *P* < 0.05. ***, *P* < 0.001. CV RR: coefficient of variation of RR. *D*, Representative recordings of A-V blocks and VT/VF at 15 and 30 μg/kg AaH-II*. *E*, Percentage of arrhythmic rats and associated phenotypes. *F*, Latency to first arrhythmia as a function of AaH-II* dose injected. *G*, Effects of AaH-II on ECG parameters in rats pre-treated with 27 μg/kg TTX. Scale bar = 200 ms. *H*, Percentage of arrhythmic rats after TTX pre-treatment. *I*, Latency to first arrhythmia at various AaH-II* doses after TTX pre-treatment. *J*, Various ECG parameters for three AaH-II* doses in rats pre-treated with 27 μg/kg TTX.

Building on our *ex vivo* results, we investigated the *in vivo* effects of AaH-II* on rat heart activity. To this aim, we first investigated the plasmatic concentrations of AaH-II* upon i.v. injection of various doses of AaH-II*. A total of 3 μg/kg AaH-II* leads to a circulating plasmatic concentration beneath 0.1 nM, i.e. the detection threshold of the mass spectrometry used (*Figure 7A*). Increasing by 5- and 10-fold, the dose of AaH-II* injected i.v. leads to plasmatic concentrations of 2.4 ± 0.4 nM (15 μg/kg) and 4.3 ± 0.6 nM (30 μg/kg), respectively. On the basis of these values, it is reasonable to assume that the AaH-II* concentration in cardiac tissue is lower than in plasma—albeit by an unknown factor. Interestingly, 3 μg/kg AaH-II* tends to produce a slight increase in the RR interval and QTc value, in spite of representing an extremely low concentration in the heart (*Figure 7B* and *7C* and *Table 2*). These effects are largely augmented at 15 and 30 μg/kg AaH-II*. For instance, the RR interval increases from 145.6 ± 2.5 ms to 181.6 ± 2.7 ms at 15 μg/kg, which is believed to be due to AaH-II* effects on sympathetic innervation (*Figure 7C*). This may also explain the increase in the coefficient of variation for the RR interval. More interesting was the observation that the QTc value increases from 46.1 ± 1.3 to 79.2 ± 4.6, which is supported by the increase in the T wave area from 1.2 ± 0.2 to 3.2 ± 0.5 mV/ms. This T wave area was further increased at doses higher than 15 μg/kg AaH-II* (*Figure 7C*). Other ECG parameters are not affected (*Table 2*). At 3 μg/kg AaH-II*, none of the rats were arrhythmic, but all became arrhythmic if higher doses were used (*Figure 7D* and *7E*). At 15 μg/kg AaH-II*, atrio-ventricular (A-V) blocks and/or isolated PVCs were monitored in all rats. At 30 μg/kg AaH-II*, all rats presented arrhythmogenic events (A-V blocks/PVCs), with episodes of ventricular tachycardia (VT)/ventricular fibrillation (VF) in half of them. The latency of appearance of arrhythmia was decreased upon increasing the dose of AaH-II* from 15 to 30 μg/kg (*Figure 7F*). Again, we cannot fully exclude that part of these observations can be explained by an indirect effect of AaH-II* on the sympathetic system through adrenergic-mediated phosphorylation of Na_v_1.5 that promoted I_NaL_. Next, we investigated how blocking TTX-S Na_v_ channels would prevent these arrhythmias. First, we evaluated the effect of various TTX i.v. doses from 3 to 243 μg/kg on ECG parameters (see Supplementary material online, *Figure S15A* and *S15B* and *Table 2*). Consistent with the effect of TTX in *ex vivo* experiments (see Supplementary material online, *Figure S14*), TTX increases the RR interval progressively with increasing doses. Interestingly, the QRS duration was not affected for doses up to 27 μg/kg, but suddenly increased at 81 μg/kg, indicating that doses above 27 μg/kg affect Na_v_1.5 activity. For these reasons, we decided to work at a TTX dose equal to 27 μg/kg, which corresponds to a circulating TTX plasmatic concentration of 403 ± 44 nM (see Supplementary material online, *Figure S15C*). While this value is four-fold higher than the concentration that should be used to be fully selective for TTX-S Na_v_ channels, we also expect that the intracardiac TTX concentration is lower in reality by the same order of magnitude. This concentration is also 25- to 75-fold lower than the one needed to block Na_v_1.5 (frequently, 10 or 30 μM are used^35^). Importantly, rats pre-treated with 27 μg/kg TTX are clearly non-arrhythmic (*Figure 7H*). In these experimental conditions, TTX prevented the appearance of VT/VF events at the various AaH-II* doses (*Figure 7H*). Only A-V blocks and PVCs were monitored, but with a clear reduction in the percentage of rats affected (*Figure 7H* and *7I*). Finally, in addition to preventing arrhythmias, inhibition of TTX-S Na_v_ channels normalized all ECG parameters with the exception of the RR interval (*Figure 7J*). There is no longer a significant difference in the RR interval between 3 and 30 μg/kg AaH-II* on the RR interval contrary to what was observed in the absence of TTX (*Figure 7C*). We assume that TTX blocks the sympathetic effects of AaH-II*. The bradycardic effects of TTX alone are coherent with what is observed *ex vivo* (see Supplementary material online, *Figure S14*) and are even more pronounced, possibly due to the combination of an intrinsic cardiac and sympathetic effect. These results indicate that, despite the fact that AaH-II* doses of 15 and 30 μg/kg potentially activate I_NaL_ from Na_v_1.5, inhibition of TTX-S Na_v_ channels has remarkable anti-arrhythmic properties.

**Table 2:** ECG parameters following AaH-II* or TTX + AaH-II* intravenous injection in anaesthetized rats

	*n*	RR (ms)	*P* wave (ms)	PR (ms)	QRS (ms)	QTc	T wave area (mV/ms)
AaH-II*							
* Baseline*	5	144.9 ± 5.3	17.1 ± 0.7	46.7 ± 1.5	17.3 ± 0.6	44.4 ± 3.6	1.1 ± 0.4
* 3 *µ*g/kg*		153.7 ± 5.6	17.5 ± 0.3	47.5 ± 1.4	16.9 ± 1.0	54.9 ± 5.2	1.8 ± 0.5
* Baseline*	6	146.2 ± 2.5	16.4 ± 0.8	42.6 ± 2.1	16.5 ± 0.3	45.6 ± 1.3	1.2 ± 0.2
* 15 *µ*g/kg*		181.6 ± 2.7*	18.0 ± 0.8	53.2 ± 5.4*	18.0 ± 0.6*	79.2 ± 4.6*	3.2 ± 0.5*
* Baseline*	6	145.4 ± 5.7	16.7 ± 0.7	46 ± 1.3	16.6 ± 0.9	47.9 ± 2.1	1.4 ± 0.3
* 30 *µ*g/kg*		176.8 ± 12.3*	18.9 ± 0.8*	53.8 ± 2.2*	17.8 ± 0.3	69.5 ± 3.3*	8.1 ± 1.9***
TTX ± AaH-II*							
* Baseline*	5	146.4 ± 10.6	14.4 ± 1.0	40.0 ± 1.5	17.3 ± 0.4	48.9 ± 1.3	2.0 ± 0.7
* 27 *µ*g/kg TTX*		194.7 ± 9.3**	16.3 ± 1.4	48.5 ± 3.3	18.2 ± 0.7	45.9 ± 2.2	1.1 ± 0.1
* 27 *µ*g/kg TTX +* *3 *µg/kg AaH-II*		207.2 ± 9.7**	15.8 ± 1.2	51.7 ± 4.0	18.2 ± 0.4	46.5 ± 1.1	0.9 ± 0.1
* 27 *µ*g/kg TTX +* *15 *µg/kg AaH-II*		211.3 ± 10.1**	16.8 ± 2.6	52.9 ± 4.7	18.9 ± 0.4	50.1 ± 2.4	1.4 ± 0.3
* 27 *µ*g/kg TTX +* *30 *µg/kg AaH-II*		216.2 ± 10.7***	16.3 ± 2.0	54.14 ± 4.5	18.5 ± 0.2	55.0 ± 2.8	1.6 ± 0.3

Wilcoxon matched-pairs signed-rank test for AaH-II* only injection. Repeated measures one-way ANOVA followed by Holm-Sidak’s multiple comparisons test for TTX ± AaH-II* injection.

*n*, number of rats.

*P*-value: *P < 0.05, **P < 0.01, ***P < 0.001 vs. baseline.

## Discussion

I_NaL_ is a well-established driver of cardiac electrical instability and a central mechanism underlying both inherited and acquired arrhythmias. Traditionally, pathological I_NaL_ has been attributed almost exclusively to dysfunction of the predominant cardiac sodium channel Na_v_1.5. In the present study, we challenge this prevailing paradigm by demonstrating that TTX-S sodium channels are not merely auxiliary contributors to cardiac excitability but are sufficient on their own to generate arrhythmogenic I_NaL_ and trigger ventricular arrhythmias. By rigorously defining the isoform selectivity of the widely used toxin ATX-II and identifying AaH-II* as a high-affinity and selective activator of TTX-S sodium channels, our work provides both conceptual and methodological advances that redefine the cellular origin of pathological I_NaL_.

### TTX-S sodium channels as independent drivers of arrhythmogenic I_NaL_

Although Na_v_1.5 accounts for the majority of peak sodium current in cardiomyocytes, accumulating evidence indicates that several TTX-S Na_v_ isoforms are expressed in the heart, with species-dependent abundance and highly specific subcellular localization. Our findings extend this knowledge by showing that selective activation of TTX-S channels is sufficient to induce a pathological I_NaL_ capable of disrupting Ca^2+^ homeostasis, prolonging repolarization, and triggering arrhythmias in native cardiac tissue and *in vivo*. Importantly, these arrhythmogenic effects occur under conditions where Na_v_1.5-mediated I_NaL_ is negligible and are abolished by nanomolar concentrations of tetrodotoxin that spare Na_v_1.5, demonstrating a necessary and sufficient role for TTX-S channels.

These results underscore a key principle: arrhythmogenicity does not scale simply with the magnitude of sodium influx but rather with its timing, kinetics, and subcellular origin. TTX-S Na_v_ channels, enriched within transverse tubules,^42,43^ are ideally positioned to elevate local Na^+^ concentrations in restricted microdomains that tightly couple to Ca^2+^-handling machinery. Even modest late Na^+^ entry through these channels can therefore disproportionately perturb excitation-contraction coupling, promote sarcoplasmic reticulum Ca^2+^ overload, and favour spontaneous Ca^2+^ release events that trigger early afterdepolarizations.

### Revisiting ATX-II-based interpretations of cardiac I_NaL_

ATX-II has long been advocated, including by regulatory agencies, as a reference tool to investigate pathological I_NaL_. However, its isoform selectivity has remained poorly defined. By performing a standardized, side-by-side functional profiling of human Na_v_ isoforms, we demonstrate that ATX-II has the ability to enhance late sodium current through both TTX-resistant Na_v_1.5 and multiple TTX-S channels if the concentration used is not tightly monitored. These findings do not invalidate ATX-II as an experimental tool but necessitate a reinterpretation of its effects: arrhythmogenic phenotypes induced by ATX-II at higher concentrations likely reflect the combined activation of several sodium channel populations rather than isolated Na_v_1.5 dysfunction.

In contrast, AaH-II* emerges as a complementary and, in some contexts, more informative pharmacological probe. Its high affinity and functional selectivity for TTX-S channels allow, for the first time, the dissection of TTX-S-dependent I_NaL_ without confounding activation of Na_v_1.5. This distinction is critical, as it reveals that a substantial fraction of arrhythmogenic late sodium current can arise independently of the canonical cardiac sodium channel.

### Relationship between Na_v_1.5 and TTX-S channels in arrhythmogenesis

Our data do not argue against a role for Na_v_1.5 in arrhythmia generation. Instead, they support a model in which Na_v_1.5- and TTX-S-mediated I_NaL_ represent complementary and potentially synergistic mechanisms. In many pathological settings, modest Na_v_1.5 late current may be insufficient on its own to destabilize Ca^2+^ handling, but amplification through TTX-S channels—particularly within t-tubular microdomains—may push the system beyond a proarrhythmic threshold. This framework reconciles apparently conflicting reports in the literature regarding the necessity of TTX-S channels for excitation-contraction coupling under physiological conditions while highlighting their critical contribution under pathological stress.

### Implications for cardiac safety pharmacology and therapy

These findings have important translational implications. Current cardiac safety paradigms, including the CiPA, largely focus on Na_v_1.5-mediated I_NaL_. Our results suggest that selective modulation of TTX-S sodium channels could significantly influence arrhythmia susceptibility without overt effects on conduction velocity or peak sodium current. Incorporating TTX-S channel assessment into preclinical screening pipelines may, therefore, improve prediction of proarrhythmic liability and help explain arrhythmias observed in neurological and neuromuscular disorders where TTX-S Na_v_ channels are primarily affected.^44,45^

From a therapeutic perspective, TTX-S channels represent attractive and potentially safer targets for antiarrhythmic intervention,^46,47^ an issue to be considered in the recently published practical compendium of antiarrhythmic drugs.^48^ Selective inhibition of TTX-S-derived I_NaL_ could attenuate arrhythmogenic Ca^2+^ signalling while sparing Na_v_1.5-dependent impulse propagation, reducing the risk of conduction block that has plagued many sodium channel-targeting antiarrhythmic drugs.

### Limitations and future directions

Species-dependent differences in the expression and distribution of TTX-S Na_v_ isoforms must be considered when extrapolating these findings to the human heart. While human induced pluripotent stem cell-derived cardiomyocytes provide valuable insight into isoform expression and pharmacological sensitivity, further studies in adult human ventricular tissue will be essential to define isoform-specific contributions in health and disease. Future work should also aim to identify which individual TTX-S isoforms—such as Na_v_1.1 or Na_v_1.6^43^—represent the most tractable therapeutic targets and to determine how their regulation is altered in specific arrhythmia syndromes.^47^

## Conclusions

In summary, this study demonstrates that TTX-S sodium channels constitute a previously underappreciated and independent source of arrhythmogenic late Na^+^ current in the heart. By introducing AaH-II* as a selective pharmacological tool and redefining the interpretation of ATX-II-induced effects, our work reshapes the conceptual framework of cardiac I_NaL_ and opens new avenues for mechanism-based anti-arrhythmic strategies that extend beyond Na_v_1.5.

## Supplementary Material

euag249_Supplementary_Data

## Data Availability

The data underlying this article are available in the article and in its online supplementary data, as well as in the following repository: https://github.com/jerome-montnach/AaH-II_arrhythmias.
