# Supplementary material for: Selective activation of tetrodotoxin-sensitive sodium channels by AaH-II drives arrhythmogenic late Na+ current in the heart
Source: Europace. 2026 Sep 3;28(9):euag249. doi: 10.1093/europace/euag249 (PMC13598332; doi:10.1093/europace/euag249)
Supplement: Supplementary file 1 [file euag249_supplementary_data.docx]

**Selective Activation of Tetrodotoxin-Sensitive Sodium Channels by AaH-II Drives Arrhythmogenic Late Na^+^ Current in the Heart**

Hugo Millet^1^, Maureen Choteau-Bodor^1^, Thomas Stervinou^1^, Floriane Bibault^1^, Sarah Godin^1^, Pauline Belhumeur^1^, Matthias Dereli^1^, Agnès Hivonnait^1^, Morteza Erfanian^1^, Agnès Tessier^1^, Aurore Girardeau^1^, Guillaume Lamirault^1^, Flavien Charpentier^1^, Mikael Croyal^1,2^, Nathalie Gaborit^1^, Jérôme Montnach^1*^, Michel De Waard^1,3*^

^1^ Nantes Université, CNRS, INSERM, l’institut du thorax, F-44000 Nantes, France.

^2^ Nantes Université, CHU Nantes, Inserm, CNRS, SFR Santé, Inserm UMS 016, CNRS UMS 3556, Nantes, France.

^3^ Laboratory of Excellence “Ion Channels, Science and Therapeutics”, Valbonne, France.

**Figure S1**. Pharmacological equivalence between AaH-II and AaH-II*. AaH-II* presents a R to K substitution at amino acid position 62. ***a***, Representative current traces of hNa_v_1.2 before and after application of 0.1 or 10 nM AaH-II or AaH-II*. Scale bars: 0.5 nA and 10 ms. ***b***, Average normalized dose-response curves showing the effect of AaH-II and AaH-II* on the late hNa_v_1.2 current (materialized by measuring AUC). Fit of the data yield EC_50_ values of 0.11 nM (AaH-II, n=52 cells) and 0.16 nM (AaH-II*, n=57 cells), respectively. Please note that above 1 nM, AaH-II leads to peak current inhibition (see arrow in panel ***a***) that is not observed with AaH-II* and that is thought to occur by binding on a second lower affinity site on voltage sensor domain I [7].

**Figure S2**. **Effect of 10 nM AaH-II* on hNa_v_1.1 channel properties.** ***a***, Representative traces of hNa_v_1.1 recorded under control condition (black trace) and after application of 10 nM AaH-II* (pink trace). Inset: enlarged view of the late sodium current remaining after a 1-s depolarization step at 0 mV. ***b***, Quantification of normalized peak current amplitude, fold change in the area under the curve (AUC) and fold change in late current. ***c***, Average voltage-current (I-V) relationships under control condition (white symbols) and after 10 nM AaH-II* application (pink symbols). ***d***, Steady-state activation and inactivation curves under control conditions (white symbols) and after 10 nM AaH-II* application (pink symbols). ***e***, Quantification of half-activation and half-inactivation potentials, as well as activation and inactivation slope factors, under control conditions (white bars) and after 10 nM AaH-II* application (pink bars). ***f***, Recovery from inactivation assessed as the P2/P1 ratio under control conditions (white symbols) and after 10 nM AaH-II* application (pink symbols). ***g***, Quantification of the fast and slow time constants of recovery from inactivation. ***h,*** Quantification of the relative contribution of the fast and slow recovery components under control conditions (black and white bar) and after 10 nM AaH-II* application (pink and red bar). Data are presented as mean ± SEM. *p < 0.05, **p < 0.01, ***p < 0.001.

**Figure S3**. **Effect of 10 nM AaH-II* on hNa_v_1.2 channel properties. Same legend as Figure S2.**

**Figure S4**. **Effect of 10 nM AaH-II* on hNa_v_1.3 channel properties. Same legend as Figure S2.**

**Figure S5**. **Effect of 10 nM AaH-II* on hNa_v_1.4 channel properties. Same legend as Figure S2.**

**Figure S6**. **Effect of 10 nM AaH-II* on hNa_v_1.5 channel properties. Same legend as Figure S2.**

**Figure S7**. **Effect of 10 nM AaH-II* on hNa_v_1.6 channel properties. Same legend as Figure S2.**

**Figure S8**. **Effect of 10 nM AaH-II* on hNa_v_1.7 channel properties. Same legend as Figure S2.**

**Figure S9**. **Effect of 10 nM AaH-II* on hNa_v_1.5 current recorded by manual patch-clamp**. The data confirm those obtained by automated patch-clamp experiments in **Figure S6**: absence of effect on peak current amplitude or I_NaL_ measured at 1 sec depolarization.

**Figure S10**. I_NaL_ induction by ATX-II on various hNa_v_ isoforms. ***a***, Representative normalized current traces elicited at test potential 0 mV before and after application of 0.1, 0.3 and 1 nM ATX-II. Note that 0.1 nM ATX-II only triggers I_NaL_ from hNa_v_1.5, and that 0.3 nM starts recruiting hNa_v_1.2 I_NaL_. ***b***, Average normalized dose-response curves illustrating the potency of ATX-II on various hNa_v_ isoforms. EC_50_ values obtained by the fit of the data are provided in **Table 1**. ***c***, Maximal level of I_NaL_ triggered by ATX-II. hNa_v_1.6 and hNa_v_1.7 are insensitive to ATX-II action.

**Figure S11**. Preserved TTX sensitivity of AaH-II*-bound-hNa_v_1.6. ***a***, Representative Na^+^ current mediated by hNa_v_1.6 at 0 mV test potential in control condition, after application of 10 nM AaH-II* to induce I_NaL_, and after application of 100 nM TTX. Scale bars: 2 nA, 10 ms. ***b***, Representative time course illustrating the amplitude of hNa_v_1.6 Na^+^ currents before and during successive applications of 10 nM AaH-II* first, and 10 nM AaH-II* + 100 nM TTX after. ***c***, Average current amplitude (left) and AUC (right) illustrating the effect of AaH-II* and of TTX normalized to control condition (n=9 cells). ***d***, Representative Na^+^ current mediated by hNa_v_1.6 at 0 mV test potential in control condition, after application of 100 nM TTX to fully block the current, and after application of 100 nM TTX + 10 nM AaH-II*. Scale bars: 1 nA, 10 ms. ***e***, Representative time course illustrating the peak current amplitude of hNa_v_1.6 Na^+^ currents before and during successive applications of 100 nM TTX first, and 100 nM TTX + 10 nM AaH-II* after. ***f***, Average current amplitude illustrating the effect of 100 nM TTX and of 10 nM AaH-II* normalized to control condition (n=10 cells). ***, p<0.001.

**Figure S12**. Proarrhythmic effects of ATX-II on adult rat ventricular cardiomyocytes. ***a***, Calcium transients triggered by electrical stimulation at 0.5 Hz in control conditions and after application of 0.1, 1 or 3 nM ATX-II. Abnormal spontaneous calcium release (SCR) events are indicated by arrows. Scale bars: 1 F/F_0_, 2 sec. ***b***, Percentage of cells that display SCR at various ATX-II concentrations. ***c***, Number of SCR events per min of recording at various ATX-II concentrations. ***d***, Amplitude and time to peak quantification of normal calcium transients before and after 0.1, 1 or 3 nM ATX-II application.

**Figure S13**. AaH-II* and TTX effects on conduction velocity parameters. ***a***, Quantification of conduction velocity in various pharmacological conditions. ***b***, Quantification of absolute inhomogeneity of conduction velocity in various pharmacological conditions.

**Figure S14**. Bradycardic effect of TTX at a concentration that does not affect Na_v_1.5. ***a***, Representative MEA recording in control and after perfusion of 100 nM TTX in isolated rat heart. Scale bar: 200 ms. ***b***, Resulting activation map. ***c***, Average interbeat interval and the modification induced by 100 nM TTX. ***, *P*<0.001. ***d***, Activation time and conduction velocity unaffected by 100 nM TTX.

**Figure S15**. Effect of increasing *in vivo* doses of TTX on ECG parameters. Representative ECG recordings in control condition and at various doses of TTX. Scale bar: 200 ms. ***b***, Average RR interval and QRS duration at various conditions TTX doses compared to control condition. ***c***, Plasmatic concentration of TTX at the dose of 27 μg/kg.

**Table S1.** Half-potential values for activation and inactivation of hNa_v_1.1 to hNa_v_1.7 channels in the absence or presence of 10 nM AaH-II* extracted from data shown in **Figure S2** to **S8**. * p<0.05 *versus* Control ; *** p< 0.001 *versus* Control.
